# Evaluating the impact of COVID-19 protection measures and staff absence on radiotherapy practice: A simulation study

**DOI:** 10.1371/journal.pone.0314190

**Published:** 2025-01-16

**Authors:** Elisabeth Jambor, Joe Viana, Melanie Reuter-Oppermann, Ralf Müller-Polyzou

**Affiliations:** 1 University of Kaiserslautern-Landau (RPTU), Kaiserslautern, Germany; 2 Department of Accounting and Operations Management, BI Norwegian Business School, Oslo, Norway; 3 Department of Health, Care and Public Health Research Institute (CAPHRI), Maastricht University, Maastricht, The Netherlands; 4 Faculty of Management and Technology, Leuphana University, Lüneburg, Germany; 5 Department of Industrial Economics and Technology Management, Faculty of Economics and Management, NTNU – Norwegian University of Science and Technology, Trondheim, Norway; 6 Center for Service Innovation, St. Olav’s Hospital, Trondheim, Norway; London School of Hygiene & Tropical Medicine Centre of Global Change and Health: London School of Hygiene & Tropical Medicine, UNITED KINGDOM OF GREAT BRITAIN AND NORTHERN IRELAND

## Abstract

**Background:**

Radiotherapy practice for cancer treatment is resource-intensive and demands optimised processes for patient throughput while guaranteeing the quality and safety of the therapy. With the COVID-19 pandemic, ad-hoc changes in the operation of radiotherapy centres became necessary to protect patients and staff. This simulation study aimed to quantify the impact of designated COVID-19 protection measures and pandemic-related staff absence on patient waiting times and throughput. The approach also enables analysis of protective measures and process adjustments for future business disruptions.

**Methods:**

A discrete event simulation model of a stand-alone radiotherapy centre was developed and used to analyse changes in patient flow when implementing COVID-19 protection measures and experiencing staff absence. The simulation results support business continuity planning and decision-making in radiotherapy. In total, twenty-one scenarios in three categories were analysed. Category 1 scenarios investigated the effect of healthcare staff and equipment shortfalls. Category 2 scenarios simulated the impact of additional COVID-19 protection measures at low COVID-19 incidence rates, while category 3 scenarios evaluated the changes at high incidence rates.

**Results:**

The simulation results suggested increased patient waiting times when staff is absent. Most scenarios of the three categories behave similarly despite increased patient waiting times due to COVID-19 protection measures in categories 2 and 3. The most significant increase in patient waiting times occurs when only two radiation therapists are available. The absence of a linear accelerator for cancer treatment also leads to increased waiting times. Scenarios where one administrator is absent show the longest average and maximum waiting times for low COVID-19 incidence rates. COVID-19 protection measures reduce patient throughput. In all scenarios, with reduced patient throughput, follow-up radiation appointments were affected.

**Conclusions:**

The simulated scenario results suggest that appropriate staffing of the radiotherapy centre during a pandemic crisis is essential and that staff absence can lead to prolonged patient waiting times and reduced throughput with severe continuity of care consequences. The simulation model demonstrated that centre administrators are a bottleneck if they must perform COVID-19 protection measures in addition to their administrative duties. The effect could be mitigated by outsourcing COVID-19 protection tasks to external service providers or other centre staff.

## Introduction

### Context

Almost 50% of people in Germany develop cancer during their lifetime, and timely diagnosis and treatment are strongly associated with recovery [1, 2]. It is essential that cancer patients start their treatment as early as possible and interruption of therapy is avoided to prevent tumour cell repopulation [3]. Radiotherapy is an important form of cancer treatment, used in approximately 50% of all cancer cases for both curative and palliative treatment [4]. The efficient operation of radiotherapy centres is essential, with no compromise being accepted in the effectiveness and safety of medical treatment [5]. Therefore, processes in radiotherapy centres should be well-coordinated and optimised [6]. Potential changes to existing synchronised processes must be justified and carefully planned before implementation.

The COVID-19 pandemic has challenged radiotherapy centres and hospital departments worldwide to adapt operations by implementing protection and infection control measures for patients and staff [7, 8]. Due to the sudden virus outbreak, timely planning of procedural changes was often impossible. In view of potential future disruptions, evaluating and optimising protection measures to ensure radiotherapy operation even in times of pandemics is necessary. Consequentially, procedural changes should be thoroughly investigated before implementation. Simulation models can help to analyse complex processes and to evaluate possible changes in a safe virtual environment [9, 10]. This article presents a discrete event simulation (DES) model of a radiotherapy centre developed to study and evaluate processes in the context of COVID-19. More concretely, the impact of pandemic-related staff absence, patient screening for COVID-19 symptoms and the application of protective measures during the treatment of patients suffering from COVID-19 were evaluated.

In order to address these aspects, we first provide the background on cancer and radiotherapy treatment in the context of the COVID-19 pandemic. In support of this, simulation literature and relevant results are highlighted. We then outline the methods used and introduce the DES model, its specifications and the experimental design. Afterwards, the simulation results are presented and discussed. Finally, after concluding and summarising our contribution, we envision areas for future research.

### Cancer and radiotherapy

Cancer is a life-threatening disease with immense physical, psychological, and economic implications. It is caused on a genetic level, resulting in body cells uncontrollably multiplying and often growing destructively into the body [11]. In 2018, the World Health Organization (WHO) reported 18 million new cancer cases and 9.6 million deaths worldwide, making cancer the second leading cause of death worldwide. The WHO predicts a doubling of new cancer cases by 2040 [12]. In addition to prevention, the effective treatment of existing cancer is crucial. Radiotherapy is an essential form of cancer treatment, along with surgery and chemotherapy [13]. Long waiting times for radiotherapy appointments can lead to a lower chance of survival [14]. Therefore, optimising radiotherapy processes and providing fast access to radiotherapy treatment is crucial.

### Percutaneous radiotherapy with linear accelerators

In percutaneous radiotherapy, called radiotherapy, radiation is irradiated from outside the body in the form of electrons and photons using linear accelerators (LINACs) [5, 15]. The therapy is delivered with high precision, applying the radiation dose to the tumour while sparing the surrounding healthy tissue. Since the radiation penetrates the body from the outside, it is impossible to prevent healthy tissue from being irradiated. Therefore, radiotherapy is usually performed in a series of irradiation fractions over several weeks. The time between the fractions allows the irradiated healthy cells to recover, which the cancer cells cannot do to the same extent. This leads to more damage to the tumour cells than the healthy tissue [16].

In radiotherapy, patients are irradiated using LINACs [16]. Therapy errors, for instance, due to incorrect dosing, can have life-threatening consequences. Therefore, radiotherapy must be carefully planned and delivered by qualified staff [17, 18], specifically radiation oncologists, medical physicists and radiotherapy technologists (RTTs), supported by administration staff. Radiation oncologists are physicians with additional training as a radiotherapist and qualifications in radiation protection [19]. Their tasks include examining the patients, establishing the justification of the indication, and informing patients about the risks of therapy. They are also responsible for radiation planning and treatment [20]. Medical physicists are involved in treatments with ionising radiation with an individual irradiation plan, drawing up the plan and carrying out the irradiation [21]. In addition to radiation planning, medical physicists are responsible for dosimetry, quality assurance of the equipment and radiation protection. RTTs are qualified in radiation protection and are authorised to use radiation on humans without supervision by a radiation oncologist [22]. In the context of radiotherapy, they are allowed to take computed tomography (CT) images and independently irradiate a patient according to the patient’s irradiation plan previously approved by the radiation oncologist. The administration staff complements the radiotherapy team and is responsible for patient reception and care, scheduling appointments, billing, patient transport coordination, and other administration tasks [23].

**Fig 1:**
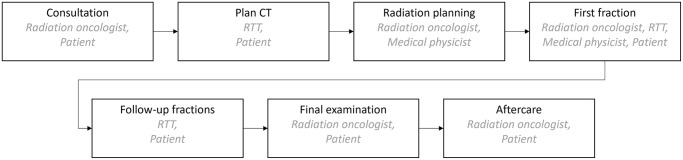
Radiotherapy process, activities and people involved.

In Germany, radiation therapy is applied in private centres and clinics as well as public hospitals. Although these centres may differ in size and equipment, the radiotherapy processes are often comparable. The course of radiotherapy can be divided into the seven main processes 1) consultation, 2) plan CT, 3) radiation planning, 4) first fraction, 5) follow-up fractions, 6) final examination and 7) aftercare, as shown in Fig 1 [13, 24]. Note that in other countries worldwide, this process might be (slightly) different, e.g. the radiation oncologist might not be present for the first treatment in all systems.

Following the cancer diagnosis and therapy decision of an interdisciplinary team of physicians and therapists, often referred to as the tumour board, the patient is referred to the radiation oncologist. In consultation with the patient, the radiation oncologist clarifies whether radiotherapy is indicated and informs the patient about the treatment. The justifiable indication by a specialist is obligatory for radiotherapy treatment [21] and is only given if the benefits of the treatment outweigh its risks. Provided that the patient agrees to the treatment, the next step is for two RTTs to perform a planning CT, which can be supplemented by additional imaging procedures such as magnetic resonance imaging (MRI). The goal is to identify the tumour’s size, form and position within the patient’s body precisely. During the planning CT, the patient is kept in the same position as during the single irradiation sessions. Based on the images obtained, the radiation oncologist and medical physicist create the irradiation plan, which defines the details of the irradiation procedure, such as tumour target volume and irradiation dose. The correct setting of the equipment is equally important as the positioning of the patient. During the initial irradiation, the RTTs, medical physicists and radiation oncologists check the patient’s positioning and settings. The RTTs perform follow-up radiation treatments. In some cases, such as high weight loss or anticipated tumour change, re-planning may be necessary and the process steps from the plan CT to irradiation treatment are repeated. A final examination by the radiation oncologist follows the last treatment session. At regular intervals, the patient will again be seen by the radiation oncologist for aftercare. Radiotherapy can also be used for special treatments, such as total body irradiation and benign diseases. Here, imaging, planning and initial setting are usually omitted.

### Radiotherapy and COVID-19

The COVID-19 pandemic affects radiotherapy procedures in many areas. In a survey of radiotherapy practice in Germany, Austria, and Switzerland 74.2% of the participating centres reported an impact of COVID-19 on their processes [25]. Most centres (54.2%) reported increased process times due to implemented protective measures as well as patients not appearing for appointments (42.5%). Additional concerns were raised about potential staff shortages caused by COVID-19 infections. These aspects can have a significant impact on the cancer patient. As previously described, radiation treatment should commence as soon as possible following the cancer diagnosis. In addition, radiotherapy treatment that has been started should not be interrupted, as this can have a detrimental effect on treatment results [3]. Even for patients infected with COVID-19, discontinuation, especially of curative radiotherapy, should be avoided subject to the patient’s health condition [26]. Continuity of care can be supported by protecting staff from infection. The same applies to cancer patients, with their higher risk of infection and expected more severe course of disease [27].

To ensure timely and complete treatment during the pandemic and to protect staff and patients from infection, different risk mitigation measures are recommended [28]. Regarding procedures, the European Society of Radiation Oncology Radiation Therapist Committee advises, among others, that face masks always be worn and that personal protective equipment be worn when handling COVID-19 patients and suspected cases. In addition, patients should be tested for fever and asked about COVID-19 symptoms at the entrance to the radiotherapy centre [29].

### Radiotherapy simulation literature

Simulation is experimentation with a model, a simplified representation of reality that accurately represents the scope and level of detail of the real system relevant to the investigation [30]. Simulations are used when experimenting with the real system is too dangerous, time-consuming, expensive, or impossible, which is often the case in the healthcare sector [30]. In the last two decades, more than 200 articles have been published that have applied DES to healthcare management, 65% of them concerning healthcare and nursing system operations [31]. Simulation methods in general and DES have also been applied to radiotherapy practice [32].

The processes of interest are usually referred to as the system and, coupled with system behaviour assumptions, form the model [9]. A simulation can be used to study the system’s behaviour under different conditions. The conditions to be changed can be integrated into the model as parameters. Thus, different scenarios can be investigated and compared. For more detailed technical information about DES refer to [9, 10] (see online S1 Appendix File 4 Simulation model STRESS documentation).

As grounding for our model, we present the results of structured literature research (SLR) reviewing radiotherapy DES models as described in the search protocol displayed in Table 1. Similarities and differences were identified and analysed. In particular, the results were evaluated in terms of model design parameters, stochastic distributions, and what-if scenarios. The relevant publications identified in the SLR are listed in Table 2 and briefly presented in the following. An extensive review of radiotherapy simulation models that exceeds what was necessary to ground our work can also be found in [33].

**Table 1:** Literature search protocol.

Section	Description
Period	Unlimited
Language	English
Strategy	Aggregative and configurative
Search keywords	Simulation methods, simulation and optimisation, radiotherapy and synonyms
Search focus	Title, abstract and keywords
Inclusion criteria	Radiotherapy with linacs, simulation, modelling of processes, empiric data
Exclusion criteria	No radiotherapy, no DES, clinical therapies, medications, particle therapy
Sources	ScienceDirect, PubMed, Scopus, expert recommendations

**Table 2:** Results from the structured literature review.

Author	Year	Title
Babashov et al. [34]	2017	Reducing Patient Waiting Times for Radiation Therapy and Improving the Treatment Planning Process: a Discrete-event Simulation Model (Radiation Treatment Planning)
Bikker et al. [35]	2015	Reducing access times for radiation treatment by aligning the doctor’s schemes.
Famiglietti et al. [36]	2017	Using Discrete-Event Simulation to Promote Quality Improvement and Efficiency in a Radiation Oncology Treatment Center.
Hosseini et al. [37]	2015	Discrete event simulation technique for evaluating performance of oncology department: a case study.
Joustra et al. [38]	2012	Reduce fluctuations in capacity to improve the accessibility of radiotherapy treatment cost-effectively.
Kapamara et al. [39]	2014	A simulation of a radiotherapy treatment system: A case study of a local cancer centre
Miranda et al. [40]	2021	Discrete-event simulation applied to a radiotherapy process: a case study of a cancer center.
Proctor et al. [41]	2007	Modelling Patient Flow in a Radiotherapy Department.
Vieira et al. [42]	2019	Improving workflow control in radiotherapy using discrete-event simulation.
Werker et al. [43]	2017	The use of discrete-event simulation modelling to improve radiotherapy planning processes.

Babashov et al. [34] used DES to examine the patient flow at the London Regional Cancer Program in Canada, from patient referral to the radiation oncologist to the start of radiotherapy. The goal was to identify bottlenecks in the scheduling process to reduce waiting times for the start of the therapy and increase patient throughput. Data from tracking software, expert interviews, and model calibrations were input values. The authors found that adding one dosimetrist had the most significant positive impact, while removing a medical physicist had the most significant negative impact on waiting time and patient flow. Other changes, such as LINAC availability or the number of radiation oncologists, only had a minor influence. The authors suggested examining the improvements concerning cancer type and urgency level and the concurrent change of resources.

Bikker et al. [35] modelled an improved scheduling of the radiation oncologists’ tasks. The newly developed weekly schedule was evaluated using DES to reduce waiting times until the first irradiation. Historical data and physician estimates were used as input data. The use of the new physician schedule reduced the patient waiting time. Larger waiting time reductions were achieved with the combination of the oncologists’ new weekly schedule and additional changes such as the elimination of specific tumour contouring time blocks. Nevertheless, none of the proposed measures were able to achieve the target waiting time. It was also proposed to investigate prioritisation rules or capacity reservations for specific patient groups.

Famiglietti et al. [36] conducted a quality improvement study of an academic radiation oncology department, for which a DES model was developed to identify inefficiencies. Data were retrieved through observation, electronic patient records, machine data and staff management software. The patient’s length of stay (LOS), the utilisation of personnel and equipment and associated costs were examined. A substantial time dependency of patient waiting times until entering the treatment room was found, which was attributed to the temporal variability of the demand and lack of mitigation measures. It was also revealed that the utilisation of staff and equipment was below 60%. The authors proposed evaluating the effectiveness of potential improvement measures in the next step.

Hosseini et al. [37] developed a DES model of a private hospital in Tehran, Iran, to identify and resolve bottlenecks in radiology and radiotherapy processes. The patient flow from entering the hospital to leaving was considered. In addition to CT and MRI imaging, radiotherapy and brachytherapy treatment were also modelled. Input data was based on staff interviews and observation over four weeks. Patient throughput and LOS were among the performance indicators evaluated. The most significant bottleneck occurred in the imaging processes by varying parameters such as number of machines or processing times. More CT or MRI devices reduced the LOS and increased the patient throughput. In contrast, adding LINAC resources showed only slightly reduced LOS.

Joustra et al. [38] investigated the processes of the radiotherapy department at an Academic Medical Center in the Netherlands for bottlenecks and analysed the patient waiting time between patient referral and the first irradiation fraction. Relevant processes were explored using a combination of queuing theory and DES. Adding a LINAC could increase the patient throughput but failed to meet throughput targets. The outpatient department was identified as the bottleneck and queuing theory was used to improve patient waiting times by reducing the fluctuations in the department’s capacity.

Kapamara et al. [39] analysed the patient flow in the radiotherapy department of two university hospitals in England belonging to the Arden Cancer Network. Their goal was to identify bottlenecks to reduce patient waiting times. The three departments of radiotherapy, brachytherapy and radionuclide therapy were modelled and simulated using data from a database, observations, and expert interviews. The four sub-processes, consultation, imaging, planning, and treatment, were modelled for the radiotherapy department. A reduction in the patient waiting times between consultation and first irradiation fraction was achieved by extending staff and machine shifts.

Miranda et al. [40] used DES and optimisation techniques to analyse the treatment processes of the radiotherapy department of the Hélio Angotti Hospital in Brazil. Model data were obtained from tracking software and an observational study. At the same time, the time the last patient left the clinic during one day and the waiting time between arrival and start of the first irradiation fraction was analysed. Thirty-two combinations of patients scheduled, treatment duration, and the possibility of double scheduling a treatment session were examined. Among others, the scheduling of the LINAC technicians and adding one LINAC were examined. The identified process and resource changes could improve productivity without affecting the quality of treatment.

Proctor et al. [41] investigated the impact of an increase in the number of patients in the radiotherapy department of the Walsgrave Hospital in England using a DES, modelling outpatient and inpatient pathways. Machine data, expert interviews and staff observations were used as model parameters. Adding additional CT or LINAC devices, increasing shift length, and treating the patient with the first available radiation oncologist reduced the patient waiting time from the first referral to the irradiation fraction when patient numbers increased.

Vieira et al. [42] used DES to evaluate the impact of push and pull strategies on patient waiting time in the research-oriented Netherlands Cancer Institute. Input data were obtained from expert interviews and historical data. The analysis revealed that the average patient waiting time increased when all patients were scheduled as push patients; however, the average number of patients treated outside a targeted waiting time decreased. The number of transfers increased with the rate of pull patients. A more even distribution of consultation appointments significantly influenced the reduction of waiting times and the number of patients who could only be treated after the waiting time target. Furthermore, eliminating pre-booked CT appointments also improved the situation.

Werker et al. [43] used DES to investigate the planning process at the Vancouver Center of the British Columbia Cancer Agency in Canada to evaluate a possible reduction of the planning time, and thus the patient waiting time for the start of the first irradiation fraction. The DES model captured tumour localisation to finalised treatment plans and utilised historical data estimates from expert interviews and surveys. Different scenarios were tested with changing resource availability. A more even distribution and shorter delays caused by radiation oncologists showed the most significant planning time reduction. The removal of an RTT caused a tremendous increase in planning time.

### Research gap and research questions

Due to the high demand for radiotherapy, a system optimised for throughput while guaranteeing the quality and safety of therapy is essential. With the COVID-19 pandemic, short-term changes in radiotherapy processes became necessary to protect patients and staff [25]. The impact of these changes on patient waiting times and throughput was often not known or challenging to estimate by the persons in charge. While current literature on radiotherapy planning generally considers resource constraints and patient throughput, hardly any publications focus on pandemic-specific challenges, such as staff absences, protective measures, and fluctuating patient volumes. While DES is widely used in the literature, to the best of our knowledge, no studies analysed the compounded effects of high COVID-19 incidence rates and demands on administrative staff, for example. This study fills this gap by evaluating the impact of pandemic-specific measures and staff shortages on patient waiting times and throughput, offering strategies for care continuity during future crises. This simulation study aimed to quantify the impact of pandemic-related staff absences and selected COVID-19 protection measures on patient waiting times and throughput in radiotherapy. Overall, it demonstrates the usefulness and applicability of DES for analysing the impact of pandemic-related measures applied to radiotherapy. Note that the simulation can also be used to analyse protection measures and process changes against future outbreaks or during especially strong flu or COVID-19 waves after the pandemic.

Consequently, the following research questions are addressed in this work:

RQ1 How do pandemic-induced protective measures and staff availability impact patient waiting times in radiotherapy practice?
RQ2 What are the critical bottlenecks in radiotherapy operations a pandemic, specifically a COVID-19 wave, and how do they affect patient throughput?

## Materials and methods

### Study design

A computer simulation study was conducted based on a DES model, to better understand and improve the operation of a stand-alone radiotherapy centre. The model utilises agents as hypothetical computer-generated patients who require radiotherapy treatment or aftercare. Each patient is assigned a unique patient ID, age, age-related walking speed, COVID-19 infection status, and a patient pathway (for more information see online S1 Appendix File 1: Model and validation and File 4: Simulation model STRESS documentation [44] in and S2 Appendix based on Generic Reporting Checklist [45]).

### Model specification

A radiotherapy centre was modelled without external dependencies. DES is suited for investigating a sequence of well-defined steps characterizing the processes in radiotherapy. Our base case model consists of two LINACs and one CT, which can be expanded for further experimentation. English was chosen as the model language for international usability, and the occupational groups were modelled independently of the country of operation, including radiation oncologists, medical physicists, RTTs and administrators. In some countries, dosimetrists support radiation planning tasks. For the purpose of this model, both are considered equivalent.

**Fig 2:**
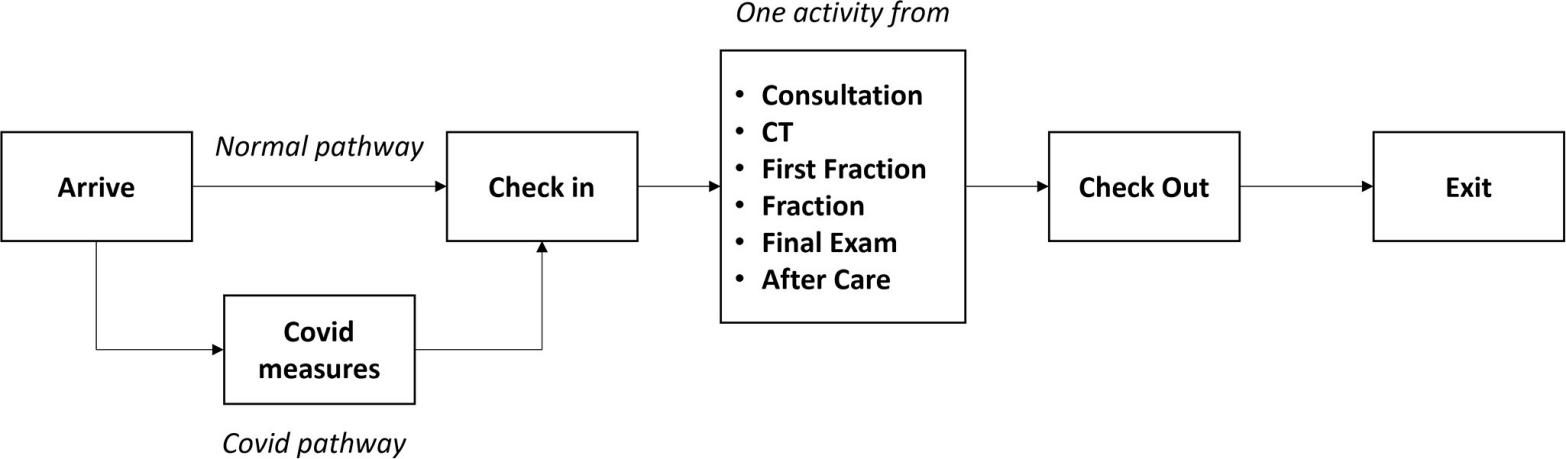
Simplified presentation of a radiotherapy centre visit.

Radiotherapy is performed in private radiotherapy centres, clinics, and public hospitals. Although these centres may differ significantly in size and technical and non-technical equipment, the general process steps of radiotherapy in Germany, as presented in Fig 1 are similar. The model focuses on the process steps that are performed on patients during a typical business day. Explicitly, patient arrival, check-in, consultation, plan CT, initial setting with first irradiation fraction, follow-up irradiation fractions, final exam and aftercare are modelled. Patient pathways based on the purpose of the visit derive the sequence of process steps in the radiotherapy centre as shown in Fig 2.

Patients arrive at the centre reception for check-in. The reception is permanently staffed with two administrators who take care of patient admission tasks and appointment scheduling. The admission tasks require more time for new consultation than for follow-up appointments. After successful admission, the patient remains in the waiting room until the treatment begins. A radiation oncologist conducts the initial consultation. Depending on the patient’s specific needs, the appointment can have a duration of 20 to 60 minutes. The radiation oncologist also performs the final examination and aftercare. The first fraction, including the initial patient setting, is performed at the LINAC by a radiation oncologist, two RTTs and one medical physicist. The challenge is to provide the necessary resources. Appointments with the radiation oncologist are time-consuming, resulting in long waiting times for patients. Thus, when the radiation oncologist is available, the remaining resources are allocated preferentially. A first fraction appointment with the initial setting requires, on average, 15 minutes. Follow-up fractions are performed at the LINAC with the support of two RTTs. The duration of the actual irradiation varies depending on the goal of the therapy. The need for special positioning aids, age, mobility, and the general state of health of the patients can prolong the irradiation session. The data collected show that a session can last between 3 and 10 minutes, with a duration of 7 minutes in most cases. Two RTTs typically perform the plan CT at the CT imaging device. Special positioning aids may also be required for this task, while the mobility status of the patient and the examination objective determine the duration in the range of 10 to 20 minutes.

At the beginning of the simulation, patients are created according to the defined distributions and provided with the attributes age, gender, treatment type, COVID-19 category and patient ID. The patients are sent to their appointments in the radiotherapy centre only if sufficient resources are available for treatment. Otherwise, the patients are sent home. If, for example, no radiation oncologist is available on the considered day, all appointments requiring a radiation oncologist must be cancelled, and the patient leaves the simulation model. Based on the average treatment duration and the need for pre- and post-work by the radiotherapy centre staff, the patient interarrival times are set to 30±5 minutes for CT scans and 10±2 minutes for follow-up radiation treatments.

In the base scenario, upon entering the radiotherapy centre, a patient proceeds to check-in. The patient must queue in case both administrators are occupied with other activities. After check-in, the patient waits in the waiting room for treatment. As soon as it is the patient’s turn and the associated resources are available, they proceed to the respective therapy room. Once the treatment or consultation is complete, the patient leaves the centre, and the resources are released for patients waiting within the centre, as per Fig 2.

In addition to the base scenario described, the present model allows the simulation of COVID-19 protection measures at the entrance to the centre. If this option is selected, the patient is instructed by an administrator to wear a face mask before entering, or they are provided with one if needed. In addition, the administrator measures the body temperature of all patients. If an elevated temperature is measured, a rapid COVID-19 test will be administered, while the administrator remains with the patient until the test result is available. The tasks of providing face masks and measuring the temperature are defined to take one minute each, while the COVID-19 rapid test requires 15 minutes.

If patients are infected with COVID-19, they will still be treated. We distinguish between patients whose COVID-19 disease was known before (from here on, *known-COVID-19 patients*) and those who test positive on the day of the treatment, either before arrival or at the entrance (from here on, *new-COVID-19* patients). An additional 30 minutes are estimated for each COVID-19 patient to allow for protective measures such as wearing personal protective equipment for staff and disinfection and ventilation of the LINAC room. For *known-COVID-19* patients, the time is accounted for in the appointment planning by delaying all subsequent patients of the affected pathway by 30 minutes. Therefore the COVID-19 measures will not add additional time to the patient waiting times, but fewer patients can be seen, thereby reducing the daily throughput of the radiotherapy centre. Also, these patients will not be subject to the COVID-19 measures at the clinic entrance and can proceed to check-in immediately, assuming that they wear face masks. For *new-COVID-19* patients, the schedule cannot be adapted, and the extra time for protection measures is not incorporated into the interarrival times. They are, therefore, disruptions to the daily plan, and additional waiting times for subsequent patients are expected. All *new-COVID-19* patients take the protection measures at the clinic entrance. The rate of patients with *known-COVID-19* is given by the prevalence (active cases minus newly infected patients of that day), and the one-day-incidence determines the rate for patients with *new-COVID-19* (newly infected patients compared to the day before) in Table 3.

**Table 3:** Low and high COVID-19 phases.

Label	Low COVID-19	High COVID-19
Date	22.10.2021	25.03.2022
New cases	19,572	296,498
Total cases	150,200	4,246,200
Rate new COVID-19	0.00024	0.00357
Rate known COVID-19	0.00157	0.0475

As low COVID-19 rates, the incidence and prevalence report from the Robert Koch Institute (RKI), the Federal Republic of Germany’s scientific institution in the field of biomedicine, dated 22.10.2021 [46] with a 7-day incidence of 95.1 COVID-19 cases per 100,000 inhabitants with 150,200 active cases (prevalence) was chosen. It was selected because a 7-day-incidence of 100 served in Germany as a guideline value for the federal emergency brake of the fourth population protection law. High COVID-19 rates were identified by the RKI report dated 25.03.2022 [47], which were the highest reported incidence values in Germany in the COVID-19 winter wave of 2021/22. The 7-day incidence rate was 1756.4 per 100,000 inhabitants, with a total of approximately 4,246,200 active cases (prevalence). The reported cases per day are displayed in Table 3.

#### Data sources

Various input data are required to represent the radiotherapy centre’s behaviour in the simulation model and to define the patients’ pathways. Furthermore, probability distributions representing the process times of the different work tasks must be defined. Additionally, patient arrival patterns and staff and equipment availability data are required. Two medical physicists at radiotherapy institutions in Germany were interviewed using a structured questionnaire to collect the required information and to reflect it with data gathered in the SLR. Participation was voluntary, and the data was anonymised. The structured questionnaire was verified by a senior medical physicist of a radiotherapy centre (for details, see online S1 Appendix, File 3: Questionnaire). The questionnaire and SLR data were supplemented by the comments of a senior RTT of a radiotherapy centre to obtain complete and meaningful model data. Thus, complemented with values derived from the SLR, probability distributions, relating to patient characteristics and process durations (service times), and parameter values could be estimated (for a detailed description of the input data and the distributions, see online S1 Appendix, File 4: Simulation model STRESS documentation).

**Fig 3:**
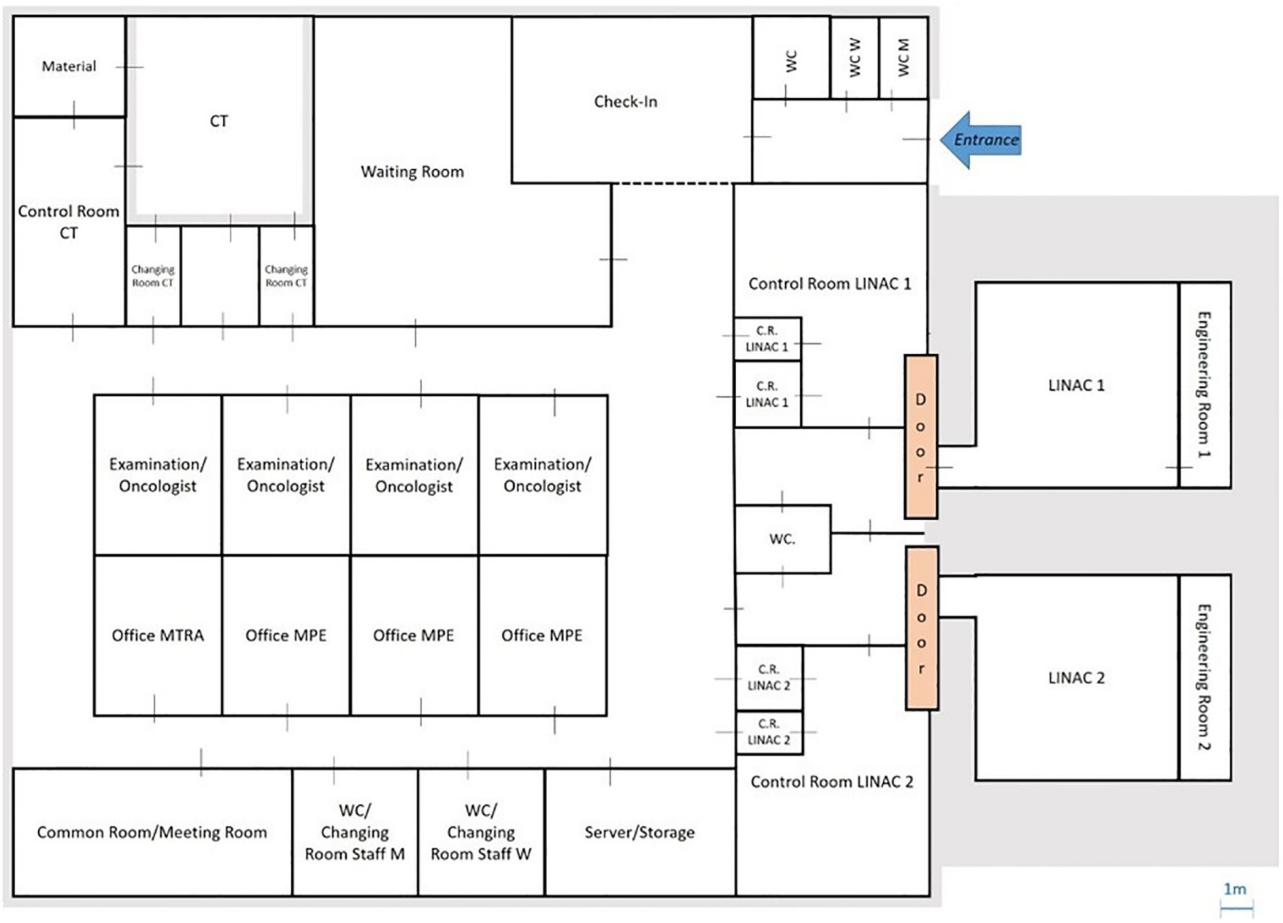
Floor plan of the radiotherapy centre model.

Concerning human resources, Full-Time Equivalents (FTEs) were assumed to work continuously during the ten-hour opening time plus an additional 30-minute grace period. Shift and break scheduling ensures the availability of the FTEs. The staff numbers indicated in the collected data were converted into FTEs for the staffing of the radiotherapy centre: 1.5 radiation oncologists, 6 RTTs, 2 administrators, and 0.1 medical physicists. For modelling purposes, the radiation oncologists and medical physicists were considered one FTE each. In radiotherapy centres, this additional time is used for treatment planning with therapy planning systems (TPS) or quality assurance (QA) of the medical devices and procedures. Regarding physical resources, two LINACs and one CT imaging device were modelled. In addition, a reception desk with two seats, a waiting room, one control room per LINAC and CT device, two rooms for radiation oncologists and a medical physicist’s office were implemented. The floor plan in Fig 3 shows the rooms of the radiotherapy centre, the corridors between the rooms and the locations of the devices.

#### Assumptions

Several assumptions were made during system modelling to capture the significant relationships and behaviours while securing the model’s transparency and simulation performance. Selected assumptions are listed below (see online S1 Appendix, File 4: Simulation model STRESS documentation for a detailed model explanation):

- The model represents one single radiotherapy centre without external dependencies.
- The model of the radiotherapy centre operates a single queue multiple server system. The First In First Out (FIFO) queuing discipline is applied in the model. Patients are not assigned to a specific resource.
- All patients are generated upon model initialisation and arrive according to their specific interarrival time and pathway.
- Unseen patients include those: i) who arrive after opening hours, ii) who are not admitted into the radiotherapy centre due to insufficient resources, and iii) who are still in the centre after operating hours.
- Medical physicists are only used during initial setting of the patient, and a conflict occurs only during two simultaneous patient settings. This scenario is unlikely, with an average of 2.5 appointments per day, leading to a maximum additional waiting time of 15 minutes.
- Implementing the COVID-19 rapid test at the centre entrance for patients with signs of fever keeps the patient and the administrators busy for 15 minutes until the test results are available.
- The treatment of COVID-19 patients is extended by 30 minutes to allow staff to put on PPE and to carry out protective disinfection and ventilation measures after treatment.

#### Verification and validation

A summary of the model verification and validation (V&V) is provided below. For more detailed information about V&V see online S1 Appendix: File 1 Model and validation contains the model and the validation spreadsheet which documents the sensitivity analysis, File 2 Results and R code, provides the raw data and code to analyse it, File 4 Simulation model STRESS documentation. S2 Appendix a Radiotherapy DES quality checklist based on [45] cross references available documents that reference V&V. In S3 Appendix a model verification and validation checklist summarises the V&V.

#### Verification

The conceptual models were co-developed and validated by system stakeholders. We utilised the visualisation and animation functionality of the simulation software [48] to conduct a structured walk-through of a patient’s visit to the centre, tracing their journey through the system. Additionally, the model was developed by author 1 who verified and validated the model with stakeholders. The model was further verified and validated by author 2 (face validity) an experienced modeller who joined the team later. Although author 2 is not independent they joined the project after the model was developed and verification and validation with stakeholders had taken place.

#### Validation

After each model development step, the model was checked for correctness, validating process time and patient distributions [9, 10, 49]. First, system time measurements were made, and the model results were compared with expected system results given the distributions and patient arrivals, external validity. Input parameters were read out after the model run and assessed against the input values to rule out the possibility of accidental change. Second, patient numbers for all treatment pathways were read out for 300 model runs. On average, 16 patients had an appointment with the radiation oncologist (expected 16). 9.03 patients received a consultation, 2.47 an initial radiation, 2.42 a final examination and 2.08 a follow-up treatment. It was demonstrated that the actual distribution of treatment steps corresponds to the specified probability distribution. Moreover, it was shown that, on average, 9.99 patients (expected 10) received CT, and 118.02 patients received follow-up irradiation. The small deviation of the patients receiving follow-up irradiation from the expected mean value of 120 occurred because not all patients could enter the centre due to the limited opening hours. Third, the simulated treatment times of the individual treatment pathways corresponded to their input distributions. The higher the number of patients per treatment pathway, the smaller the calculated variation. Thereby, the time input data was validated.

In addition, histograms were used to check treatment and waiting times, and utilisation of resources during the simulation runs. Cross validation comparing the model with other models [45] was not conducted as the context in which our generic model was applied differs from those in the literature. Predictive validation, the ability of the model to predict future events [45], was not conducted due to the model’s time scale. Extensive external and face validity were performed, and sensitivity analysis was to investigate uncertainty associated with the model parameters and structure, see online S1 Appendix File 1: Model and validation, validation spreadsheet. The sensitivity analysis focussed on extreme value tests, traces, and variations to service time distribution and appointment slots were investigated; for more information, see the online supplement.

### Experimental design

#### Time horizon

The simulation model is time agnostic and can run over any defined time horizon. The opening time of the radiotherapy center is 10 hours per day, from 8:00 to 18:00. A 30-minute grace period from 18:00 to 18:30 is assumed, during which patients who were still accepted before the center closing are allowed to finish their treatment. This period is considered acceptable overtime for the radiotherapy centre staff, while no further extension of the opening hours is allowed. Patients who have not left the radiotherapy centre by this time are considered not to have received radiotherapy treatment.

#### Multiple runs

Since the model contains the stochastic elements service times and patient characteristics, several simulation runs are needed to derive the defined key performance indicators (KPIs) waiting times and patient throughput. Consequentially, determining the required number of simulation runs requires considering these KPIs. Following an extensive graphic analysis, in which we investigate the changes in the mean for all KPIs as well as the impact on model runtime [9, 49], the number of simulation runs was set to 300 (for justification see online S1 Appendix File 4: Simulation model STRESS documentation section 4.3. Estimation approach).

#### Input parameter

Through discussion with radiotherapy experts and supported by the results of the SLR, the input parameters were defined as outlined in Table 4 (for detailed information about the input parameters see Section 4 Simulation model STRESS documentation in S1 Appendix). Subsequently, a scenario-based experimental design study was performed, which is discussed in the following sections.

**Table 4:** Scenario variables.

Group	Description	Data source
COVID-19 incidence^s^	Different incidence rates (new cases/population)	RKI [46]
COVID-19 prevalence^s^	Different prevalence rates (active-new cases/population)	RKI [47]
COVID-19 measures^s^	COVID-19 measures prior to check in (on/off)	Interview, DEGRO [28], ESTRO [29]
Staff^s^	Number of Radiation oncologists Number of Medical physicists Number of RTT Number of Administrators	Questionnaire, interview
Equipment^s^	Number of CT Number of LINAC	Questionnaire, interview
Patient attributes	Patient type and age	IARC [50], FAT [51]
Process times	Process service time distributions	Questionnaire, interview

#### Key performance indicators

The scenarios in the experimental study were evaluated using the KPIs presented in Table 5. Mean KPI values and associated confidence intervals from the scenarios are calculated and presented in the Results section.

**Table 5:** Key performance indicators.

KPI	Description
Patients treated	Patients leaving the centre are considered successfully treated and counted till the end of the business day.
Patient throughput	The number of patients treated by day.
Cumulative waiting times	The total queuing time represents the patient waiting time and can be caused by COVID-19 measures, waiting for check-in, and before treatments. Waiting times are accumulated and recorded individually for each treatment pathway.
Resource utilisation	Utilisation of the staff and the radiotherapy centre equipment.
Waiting, process and moving times	Values are recorded for each process a patient passes through based on the treatment pathway.

#### Scenarios

The simulation model scenario analysis investigated the effects of COVID-19 related changes on patient waiting times and throughput. The effects of resource shortfalls and breakdowns were investigated with and without COVID-19 protection measures implemented at low and high COVID-19 incidence rates. This results in a total of 21 scenarios, as shown in Table 6.

**Table 6:** Experimental scenarios.

Resource breakdown and shortfalls	Category 1 no protection no COVID-19	Category 2 protection low COVID-19	Category 3 protection high COVID-19
No failure	Scenario 0	Scenario 7	Scenario 14
1 oncologist	Scenario 1	Scenario 8	Scenario 15
2 RTT	Scenario 2	Scenario 9	Scenario 16
4 RTT	Scenario 3	Scenario 10	Scenario 17
1 administrator	Scenario 4	Scenario 11	Scenario 18
1 oncologist, 2 RTT, 1 administrator	Scenario 5	Scenario 12	Scenario 19
1 LINAC	Scenario 6	Scenario 13	Scenario 20

The scenarios of resource unavailability were analysed in categories 1 to 3 (see Table 6). Since two RTTs always operate one LINAC or one CT device together, only group failures are examined. First, the staff shortage in each occupation was considered individually to explore the group’s influence on the performance KPIs. Subsequently, the personnel shortfall was analysed across occupational groups.

Staff shortages and resource breakdown reduce the centres capacity. In the no-failure scenario, staff and equipment are fully functional with no downtime. In this scenario, there are two radiation oncologists at 75% each, 6 RTTs, one medical physicist at 0.1% and two administrators, one CT and two LINACs.

## Results

### Waiting times (RQ1)

**Fig 4:**
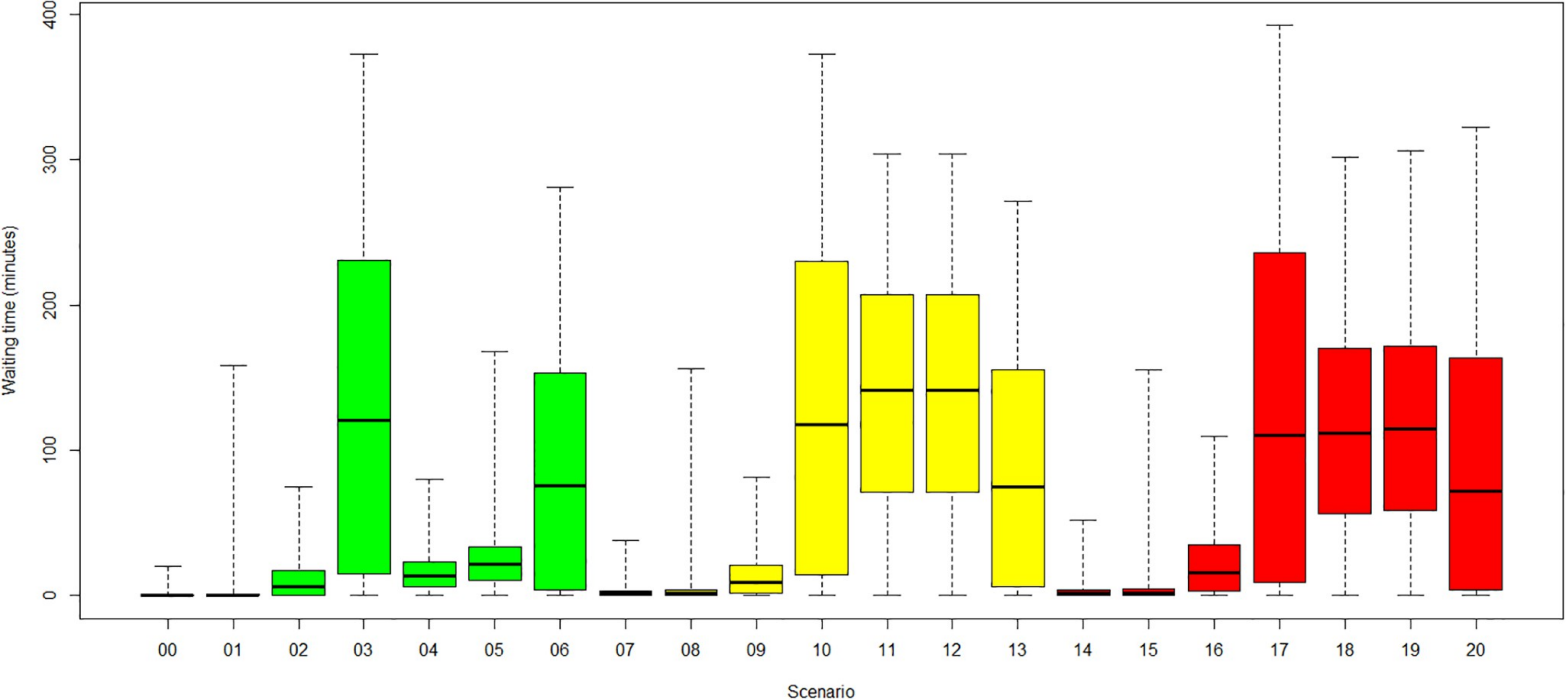
Average waiting time per scenario. (Green) Category 1: no COVID-19. (Yellow) Category 2: low COVID-19. (Red) Category 3: high COVID-19.

An overview of the average waiting times for all patients for the scenarios is presented in Fig 4.

The data presented in Fig 4 show that the waiting time is predominantly independent of the COVID-19 incidence rates for scenarios with all administrative staff available (scenarios: 0–3, 6; 7–10, 13; 14–17, 20). Although the treatment of each *new-COVID-19* patient was prolonged by 30 minutes, it resulted in a minor increase in mean waiting times due to the small number of affected patients. All patients who entered the radiotherapy centre before the first *new-COVID-19* patient are unaffected. The dispersion and the maximum of the mean waiting times increase since following patients can be strongly affected by the change in the operating procedure.

**Fig 5:**
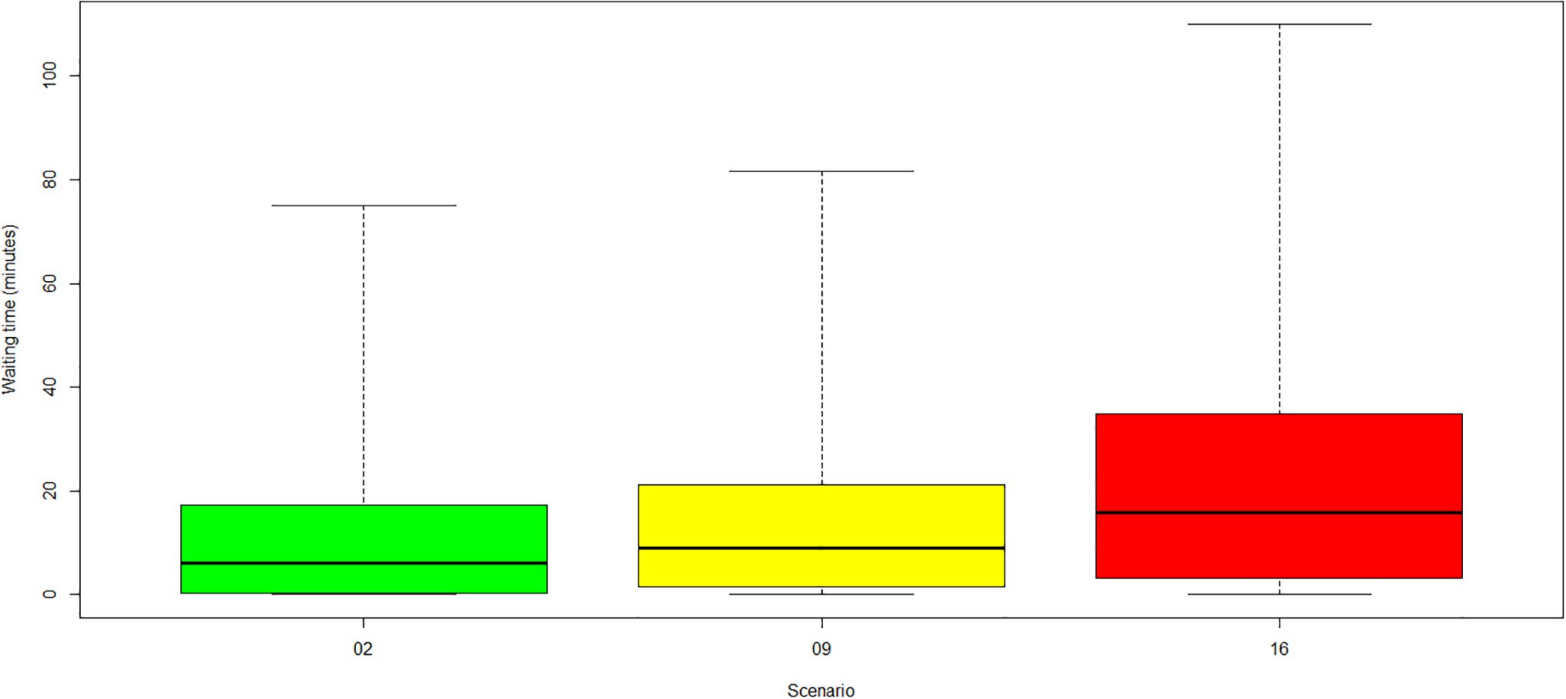
Average waiting times for scenarios missing 2 RTT. (Green) Category 1: no COVID-19. (Yellow) Category 2: low COVID-19. (Red) Category 3: high COVID-19.

The most substantial increase in waiting times arose for the scenario in which the RTT team was reduced by 4 RTTs (scenarios 3, 10, 17). The mean waiting times reached almost two hours, while the maximum waiting times exceeded even six hours. A high waiting time increase was observed in all categories in case of a LINAC failure (scenarios 6, 13, 20). Mean waiting times increased to more than one hour, and maximum waiting times reached approximately five hours. The behaviour was almost independent of the COVID-19 incidence rates and the application of related protection measures at the entrance of the radiotherapy centre. Similar behaviour was observed in scenarios with one missing radiation oncologist (scenarios 1, 8, 15). The mean and maximum waiting times were similar for all categories. For scenarios with two missing RTTs (see Fig 5), the mean and maximum waiting times increased with the COVID-19 prevalence and incidence. Each *new-COVID-19* patient (given by incidence) adds 30 minutes of treatment time, although the appointment slots were not prolonged, leading to a waiting time increase for subsequent patients. This increase was mainly experienced for follow-up radiation because of the short interarrival time between treatment appointments (10±2minutes). Also, all *known-COVID-19* patients (given by prevalence) blocked the resources for an additional 30 minutes compared to non-COVID-19 patients, thereby increasing waiting time in all other areas dependent on these resources. The reduced effect of incidence on waiting times for the scenario with four missing RTTs was due to saturation effects. The problem of four missing RTTs was already so intensive that the incidence added little further disruption.

**Fig 6:**
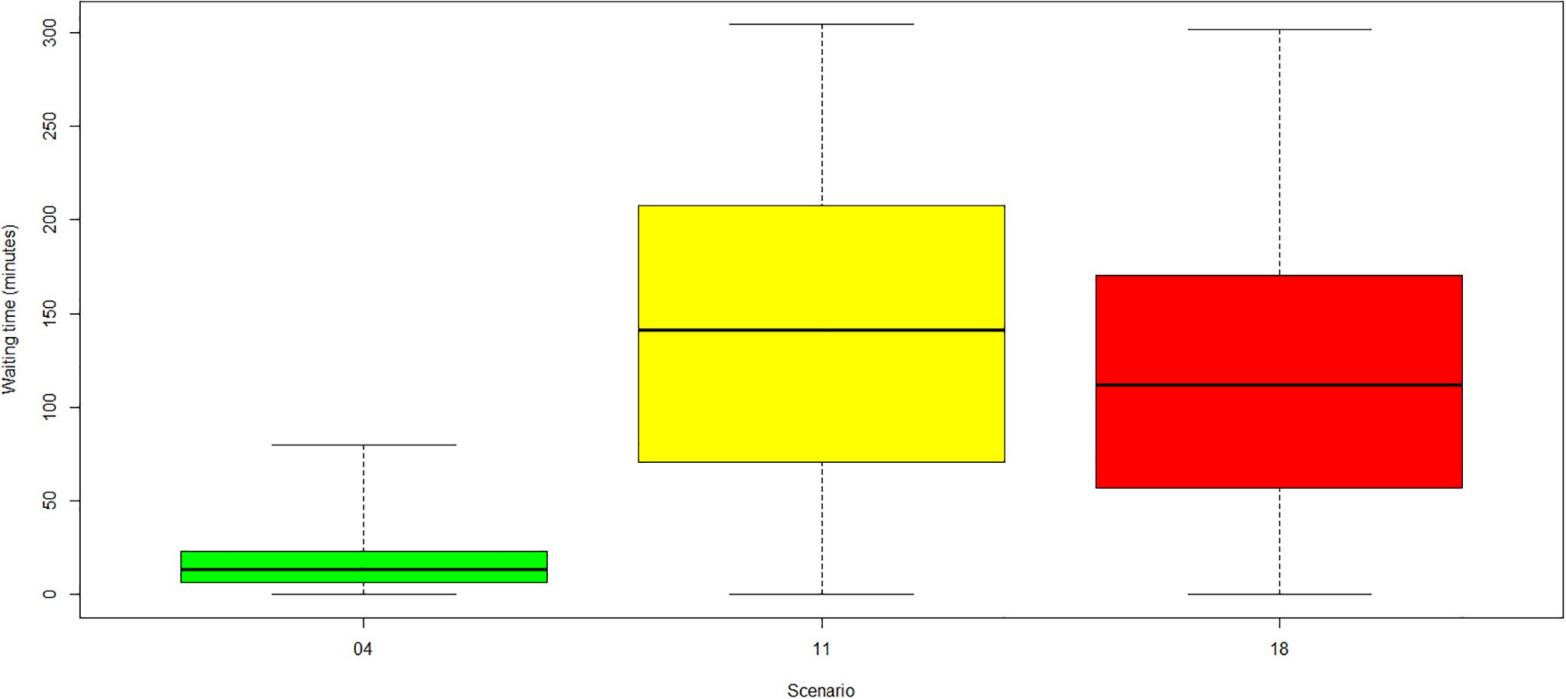
Average waiting times for scenarios missing 1 administrator. (Green) Category 1: no COVID-19. (Yellow) Category 2: low COVID-19. (Red) Category 3: high COVID-19.

**Fig 7:**
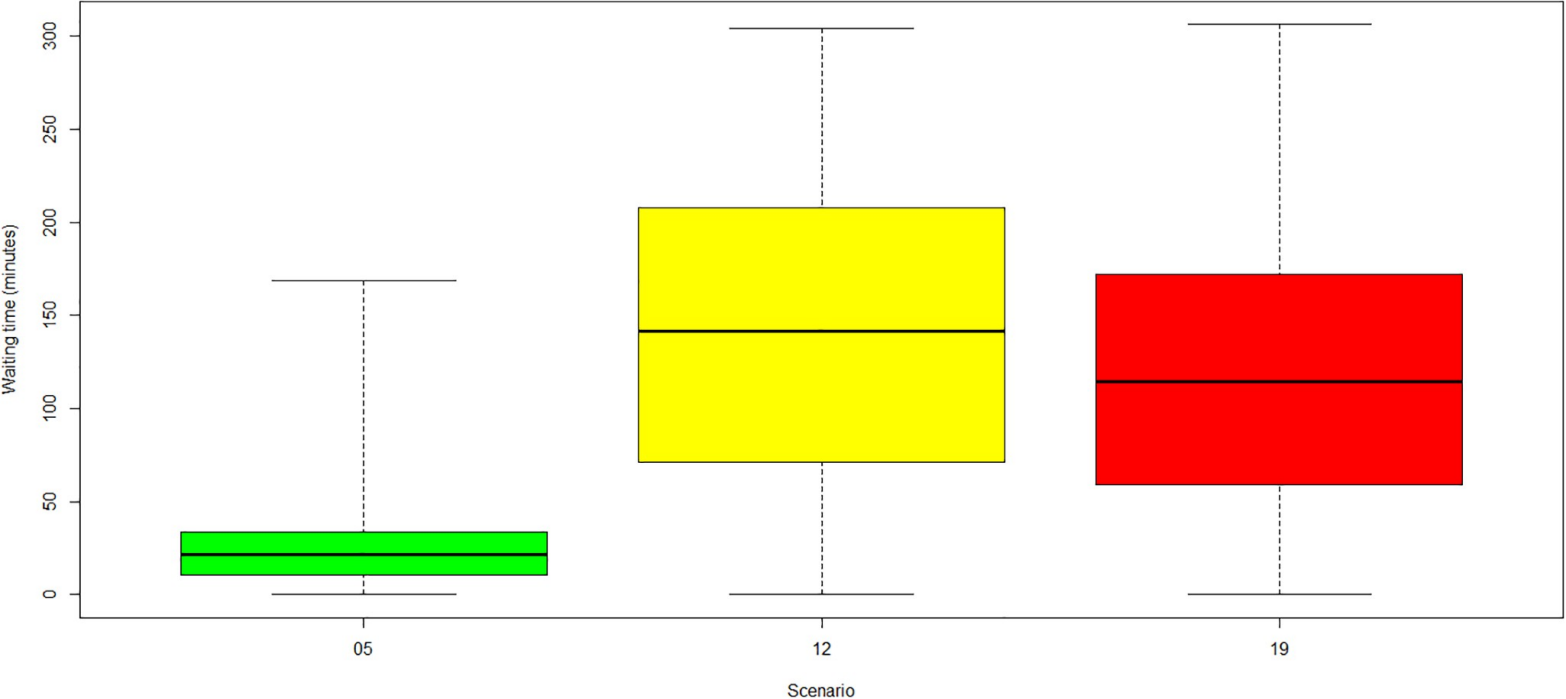
Average waiting times for scenarios missing 1 oncologist, 2 RTT and 1 administrator. (Green) Category 1: no COVID-19. (Yellow) Category 2: low COVID-19. (Red) Category 3: high COVID-19.

For scenarios 4, 11 and 18 shown in Fig 6 and scenarios 5, 12 and 19 shown in Fig 7 in which one administrator is unavailable, significant differences between the baseline case without COVID-19 and the cases with COVID-19 protection measures were observed, with highest waiting times in the low-COVID-19 category 2. The reason is that administrators must manage the COVID-19 protection measures in addition to their regular check-in tasks. All patients, except for the *known-COVID-19* infected patients, must queue for the protection measures. Afterwards, they must queue again for the check-in. The administration desk, however, is not occupied while COVID-19 measures are executed, explaining the general increase in waiting times for COVID-19 scenarios. The reduction in waiting times for the *high-COVID-19* case compared to the *low-COVID-19* case can be explained by the amount of *known-COVID-19* patients. *Known-COVID-19* patients bypass the line of the COVID-19 protection measures and go straight to the check-in. These patients wait significantly less time and reduce the queue size at the centre entrance. Also, their appointments are scheduled with 30 minutes additional time, resulting in fewer patients entering the queue at a time.

### Patient throughput (RQ2)

**Fig 8:**
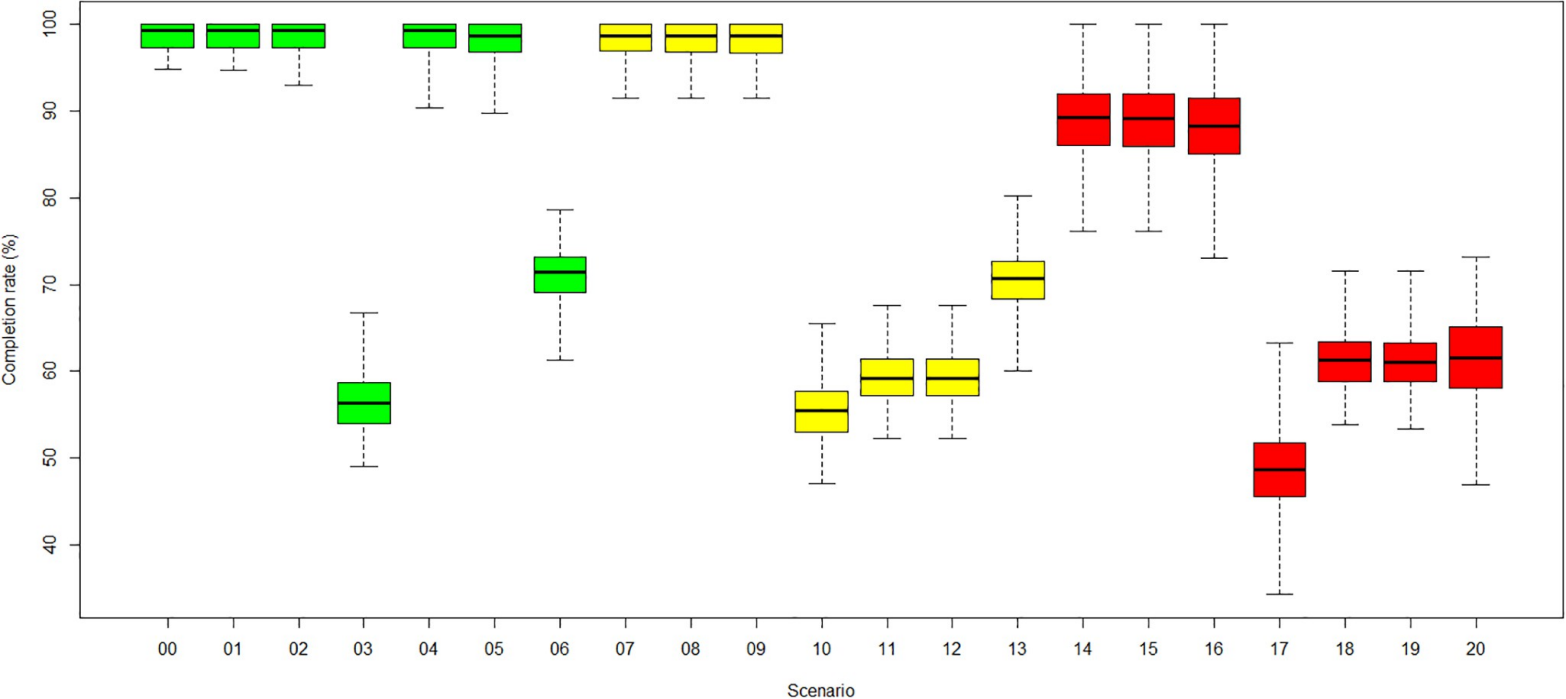
Average patient appointment completion rate per scenario. (Green) Category 1: no COVID-19. (Yellow) Category 2: low COVID-19. (Red) Category 3: high COVID-19.

An overview of the throughput, described as the number of treated patients scaled by the number of generated patients, also referred to as Patient Appointment Completion Rate (PACR), for the scenarios, is presented in Fig 8. The PACR defines patient throughput as one essential KPI of the simulation study. The scenarios with fewer resources and corresponding long patient waiting times result in a lower PACR (Fig 8). Also, planning longer treatment slots for infected COVID-19 patients reduces the number of possible appointments during one operating day.

Fig 8 shows the reduction in the PACR in three categories when 4 RTTs (scenarios 03,10,17) or 1 LINAC (scenarios 6, 13, 20) are unavailable. Lower PACR can also be observed in category 2 (scenarios 11 and 12) and 3 (scenarios 18 and 19) in the case of one unavailable administrator. Generally, in category 3, all scenarios show a reduction of the PACR of at least 10% compared to the baseline category 1, even when the radiotherapy centre is operating at total resource capacity. The reasons are that the applied COVID-19 measures prolong the appointments of COVID-19 patients by 30 minutes, leading to fewer treatment slots during a working day. This effect is most substantial for scenarios in category 3 with a high COVID-19 incidence rate. The most considerable mean reductions in PACR is observed for scenarios 3, 10 and 17 with four unavailable RTTs, with only 56% in categories 1 and 2 and 49% in category 3.

**Fig 9:**
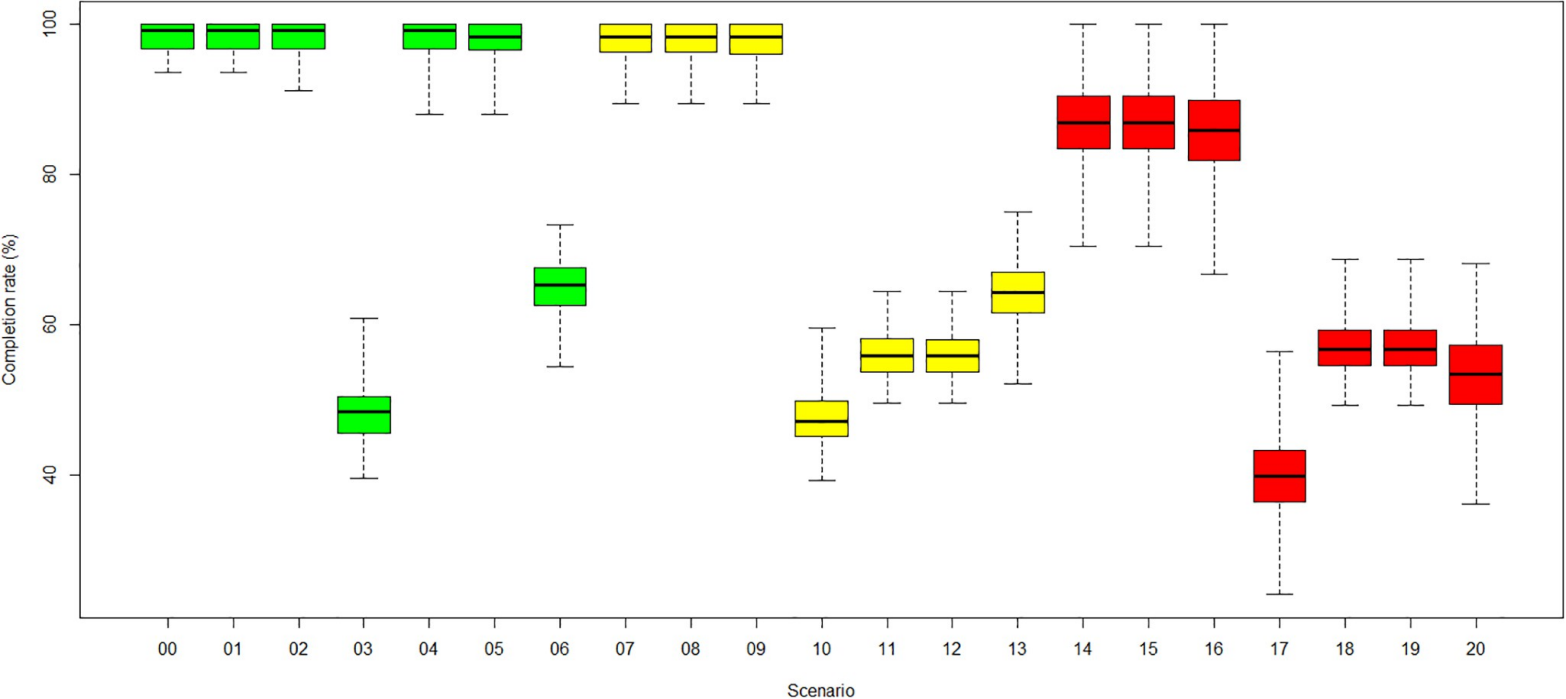
Average follow-up radiation patient appointment completion rate per scenario. (Green) Category 1: no COVID-19. (Yellow) Category 2: low COVID-19. (Red) Category 3: high COVID-19.

Fig 9 shows the PACR for patients with follow-up radiation appointments, who should not interrupt their course of fractions. These patients encounter a reduction of 52% in categories 1 and 2 and 60% in category 3 for scenarios 03, 10, 17 with four unavailable RTTs. RTTs are an essential resource shared between CT imaging and the first and the follow-up radiation appointments. If only 2 RTTs are available, only one of these treatments can be implemented at a time. This even affects patients with a radiation oncologist appointment that do not require RTT resources because they must wait for the remaining resources to become available. Due to the low number of first radiation appointments and the high priority on seizing the remaining resources as soon as they are available, this effect is moderate.

In all cases with a reduction of patient throughput, patients with follow-up radiation appointments are affected (Fig 9). For scenarios 11 and 18 with one less administrator in categories 2 and 3, patients from all other treatment branches are also affected. Patients scheduled for planning CT are also affected by the unavailability of the 4 RTTs (scenarios 3, 10 and 17) because they share the 2 remaining RTTs with the patients receiving radiation fractions.

In general, fewer patients are treated if COVID-19 protection measures are applied for low and high COVID-19 rates. Note that during the COVID waves, fewer patients might have been diagnosed and referred to radiotherapy clinics. Then, clinics might not have necessarily felt the impact of protection measures on patient throughput that much. Fig 8 shows the appointment completion percentages of the different scenarios, calculated as the number of treated patients divided by the number of generated patients, with 100% meaning all generated patients are treated and 0% meaning no patients were treated. As slightly more patients are generated than can be treated within clinic opening hours, the rate is not 100% but 98% for the base case, scenario 0. Usually, the impact is higher with increasing COVID-19 infection rates. In particular, for high COVID-19 rates, a drop of 10% occurred already in the base scenario, in which all staff and devices are available. The implementation of COVID-19 measures can explain the effect. An exception are the scenarios with 1 missing administrator (4, 11, 18), in which the patient appointment completion rates for high COVID-19 rates are more higher than the ones for low rates. This outcome aligns with the observed behaviour of the patient waiting times for these scenarios. With high COVID-19 rates, more *known-COVID-19* patients can bypass the COVID-19 protection measures queue at the centre entrance and arrive earlier for treatment. Hence, more patients can be treated within the radiotherapy centre’s opening hours.

## Discussion

### Main findings

The simulation results show that the average patient waiting times generally increased with staff unavailability. The system’s behaviour in the individual scenarios is comparable between categories 1, 2 and 3, even if the effect in categories 2 and 3 is more significant due to the additional waiting times resulting from the COVID-19 measures. Categories 2 and 3 deviate strongly from category 1 only in scenarios with a reduction to one administrator. Waiting times in categories 2 and 3 increase disproportionately since the administrator also carries out the COVID-19 protection measures at the radiotherapy centre entrance in addition to their actual tasks. The most significant increases in waiting time occur when two of the six RTTs are available (scenarios 03, 10, 17). The absence of a LINAC also leads to long average waiting times (scenarios 06, 13, 20). The average waiting times seem more acceptable if only four RTTs or one radiation oncologist is working, but the maximum waiting times are questionable. For scenarios with one available administrator, the waiting times for low COVID-19 rates are higher than for high COVID-19 rates. For high COVID-19 rates, there are more *known-COVID-19* patients whose additional treatment time is already accounted for in the scheduled treatment time and who can bypass the queue for COVID-19 measures at the radiotherapy centre entrance.

Long waiting times are a mental and physical strain for cancer patients. In radiotherapy, most patients receive radiation treatment every day for several weeks. Therefore, long waiting times should be avoided. During pandemics, long waiting times are an additional burden because it is difficult to maintain social distance in the waiting rooms to prevent infection. In addition, many patients remain in the waiting room for a long time. Thus, maintaining the minimum distance is potentially insufficient to provide adequate infection protection [47]. In addition, crowded waiting rooms also promote the transmission of other infectious diseases, which should be avoided for immunocompromised patients. At the same time, the risk of staff becoming infected increases, leading to a vicious circle of further unavailability.

Long waiting times also mean fewer patients may be treated. This outcome occurs when there is a significant reduction in RTTs when a LINAC fails and in categories 2 and 3 when an administrator is absent due to health reasons. In all cases, patients with follow-up radiation treatments are affected. In the event of a reduction in administrators in categories 2 and 3, patients from all treatment paths are affected. The lowest mean PACRs of 48% for categories 1 and 2 and 40% for category 3 are observed for follow-up radiation patients for a reduction by 4 RTT. It is essential to prevent the loss of follow-up radiotherapy, as this can lead to an accelerated repopulation of cancer cells and thus potentially lower the chances of recovery [3]. However, all other appointments must also be kept, as the patient cannot start life-saving treatment without attending a consultation or plan CT appointment. Similarly, final and follow-up examinations are essential to monitoring treatment success.

One way to reduce the loss of radiotherapy treatments could be to postpone benign radiation appointments, which would prolong and possibly worsen the suffering of these patients. It should, therefore, be considered carefully and should not be a long-lasting solution. Performing medically justifiable hypofractionation treatment schemes shortens the duration of treatment and thus frees up resources [25]. In hypofractionation treatment, higher radiation doses are delivered in fewer treatment fractions. Hypofractionation, therefore, seems to be an effective means of maintaining high patient throughput.

The simulation results suggest staffing radiotherapy centres with sufficient expert staff is essential. The analysis also indicates that staff absence can result in reduced throughput, which may lead to severe patient consequences. Accordingly, it is essential to develop solutions that ensure staff availability in radiotherapy centres. Solutions must prevent staff infection but can also address other areas. For instance, a radiotherapy centre in Germany offered childcare for the children of their staff during school closures caused by the pandemic [25]. The model identified the administrators as a potential bottleneck with far-reaching consequences for radiotherapy centre operations if they must implement COVID-19 protection measures at the centre’s entrance. This effect could be mitigated by outsourcing the tasks to external service providers. The possibility of self-registration could also be considered, reducing the workload of administrators.

To prevent waiting room congestion, selected appointments with the radiation oncologist not requiring a physical examination could be conducted using telemedicine approaches [25]. However, setting up video consultations involves time and investment in related infrastructure. Furthermore, data protection requirements must be fulfilled, and patients must be willing to accept new services.

It was shown that the impact on operating procedures caused by the COVID-19 pandemic was not caused by infected patients whose treatment was prolonged but by implementing protective measures. A reduction in the PACR could already be observed in the base scenario for high COVID-19 rates. The scenarios with low COVID-19 rates and no absence of administrators did not show a noticeable dependency on the incidence rate. For those scenarios with high COVID-19 rates, the PACR dropped. The incidence value also plays a role insofar as government-ordered closures of care facilities and quarantine obligations can lead to increased staff shortages.

### Managerial insights

As stated in the previous section on main findings, staff shortages and LINAC downtime significantly influence patients’ waiting times, proofing again the importance of sufficiently high staffing levels. Especially with increasing staff shortages, it is crucial that managers always hire sufficient staff for their radiotherapy clinics and are prepared if a pandemic or another disruption occurs.

Another main managerial insight our study provides is the value of using a mathematical simulation model to analyse processes and their potential changes in radiotherapy clinics, e.g., when installing protective measures to prepare for or cope with a COVID or a flu wave. (Note that this is true for any potentially upcoming respiratory or highly contagious disease.) A DES model can support agile decision making in a time-pressured and critical situation when guidelines from professional organisations are missing, as is was especially the case at the beginning of the COVID-19 pandemic [25].

### Strengths and limitations

The model simulates the essential processes of a radiotherapy centre. However, the model does not include processes that do not require patient presence, such as treatment planning or QA tasks. Therefore, the influences of these areas on operations are not simulated. Also, only the most widely accepted protection measures were investigated. In the present model, FTEs work in a 10-hour shift without breaks. If one examines the absence of such an FTE, the lack of several persons is effectively considered. A shift system could be modelled, representing the individual employees’ availability. Thus, FTE conversions could be avoided. Another limitation of the model is the parameter values and probability distribution functions applied. Precise centre-specific data could be collected through a Methods-Time Measurement (MTM) study, where the times required for individual processes are measured and analysed [52].

### Interpretation and further work

The SLR of radiotherapy DES simulation studies provided comprehensive data but also revealed the need for further simulation modelling of radiotherapy cancer treatment. The academic literature focused on improving the efficiency of radiotherapy processes in hospitals, while stand-alone radiotherapy centres were not considered. Furthermore, none of the scientific simulations analysed the impact of COVID-19 on radiotherapy processes. The present work closes this gap by modelling a stand-alone and internationally applicable radiotherapy centre.

The scenario analysis showed that staff shortages affect radiotherapy cancer treatment. In all occupational roles, the loss of staff results in unacceptable waiting times for patients. In some cases, the loss of staff can even lead to a drastic reduction in patient throughput. By implementing COVID-19 protection measures conducted by administrators at the entrance of the radiotherapy centre, the administrators become a bottleneck with negative consequences for the patient waiting times and throughput. The data also showed that the adverse effects depend less on the COVID-19 incident rates but more on pandemic-related staff absences and the implementation of COVID-19 protection measures by staff in addition to their existing duties.

The treatment planning and QA processes will extend the simulation model in future work, allowing us to map and systematically examine the radiotherapy workflow. The simulation model will also be used to question and verify proposed solutions and implemented changes for effectiveness and efficiency in radiotherapy. We intend to offer this possibility to all clinical users by connecting the simulation model with a Decision Support System (DSS) developed in previous work [53]. The interplay of DSS and DES simulation can help decision-makers reach fact-based decisions, particularly in crisis situations.

Evolving the simulation model into a digital twin could support agile decision-making by enabling real-time monitoring and adaptation to changing conditions, increasing resilience and efficiency in radiotherapy center operations. With live data integration, the digital twin could dynamically predict patient waiting times and resource bottlenecks and adjust operations in response to staff shortages or equipment issues.

## Conclusions

The COVID-19 pandemic posed significant challenges to radiotherapy centres worldwide. As cancer patients are at particular risk of severe COVID-19 due to their immunosuppression, they also require special protection from radiotherapy centre-acquired infection. In addition, the infection of qualified and urgently needed staff must be prevented to ensure the uninterrupted and unrestricted continuation of radiotherapy centre operations. To this end, COVID-19 protection measures are applied in radiotherapy centres. This work quantified the impact of pandemic-related staff absences and selected COVID-19 protection measures on patient waiting times and throughput in a radiotherapy centre. The results assisted radiotherapy centre decision-makers in planning radiotherapy treatment services and limit the impact of pandemic-caused care disruptions.

## Supporting information

S1 Appendix Mendeley data. 1. Model and validation. 2. Results and R code. 3. Questionnaire. 4. Simulation model STRESS documentation.
(PDF)

S2 Appendix Radiotherapy DES quality checklist. Additional model documentation.
(PDF)

S3 Appendix Model verification and validation checklist. A check list of verification and validation tests performed. This document supplements the 1. Model and Validation and 2. STRESS documentation from S1 and S2 Appendices.
(PDF)
