# Supplementary material for: Evaluating the impact of COVID-19 protection measures and staff absence on radiotherapy practice: A simulation study
Source: PLoS One. 2025 Jan 16;20(1):e0314190. doi: 10.1371/journal.pone.0314190 (PMC11737702; doi:10.1371/journal.pone.0314190)
Supplement: S1 Appendix — 1. Model and validation. 2. Results and R code. 3. Questionnaire. 4. Simulation model STRESS documentation. (PDF) [file pone.0314190.s001.pdf]

S1 – Mendeley Data

We have submitted the following to Mendeley Data:

File	Description
S1.1 Model and validation	Folder containing the AnyLogic Simulation model and the validation tests.
S1.2 Results and R code	The simulation scenario raw results, validation analysis simulation model results<br>R code to analysis simulation model output
S1.3 Questionnaire.docx	Anonymized questionnaire to ascertain quantitative and qualitative information about radiotherapy centres.
S1.4 Radiotherapy model STRESS documentation.docx	Simulation model documentation.

Jambor, Elisabeth; Viana, Joe; Reuter-Oppermann, Melanie; Müller-Polyzou, Ralf (2023), “Radiotherapy practice simulation model”, Mendeley Data, V1, doi: 10.17632/7h8t38yp9z.1

https://data.mendeley.com/datasets/7h8t38yp9z/1
