# Supplementary material for: Evaluating the impact of COVID-19 protection measures and staff absence on radiotherapy practice: A simulation study
Source: PLoS One. 2025 Jan 16;20(1):e0314190. doi: 10.1371/journal.pone.0314190 (PMC11737702; doi:10.1371/journal.pone.0314190)
Supplement: S2 Appendix — Additional model documentation. (PDF) [file pone.0314190.s002.pdf]

Reporting Quality of Discrete Event Simulations in Healthcare

This document is based on Table 1 from Zhang, X., Lhachimi, S. K., & Rogowski, W. H. (2020). Reporting Quality of Discrete Event Simulations in Healthcare–Results From a Generic Reporting Checklist. *Value Health*, *23*(4), 506-514. https://doi.org/10.1016/j.jval.2020.01.005 .

The checklist is based on good practice simulation modelling guidance developed by the International Society of Pharmacoeconomics and Outcomes Research (ISPOR) and the Society for Medical Decision Marking (SMDM) [1, 2]. The good research practice was published in a series of seven reports, the roman numerals under the checklist items below relate to the supporting best practices from this series. Note that the ISPOR-SMDM papers referenced suggest 99 best practices if subcomponents and specific examples are included. This checklist references 34 of the 99 practices.

Where links to existing documentation exist, references are provided in response to the questions rather than repeating the material. This proposed generic checklist relates to discrete event simulation (DES) models so other simulation methods (System Dynamics, Agent Based Modelling, Monte Carlo Simulation, Markov Modelling) or DES combined with other approaches may not correspond well to the checklist questions.

Model conceptualization

1. Is the focused health-related decision problem clarified?

… the decision problem under investigation was defined. DES studies included different types of decision problems, e.g., those listed in previously developed taxonomies.[3 ,4 ,5, 6, 7]

ISPOR-SMDM Modelling Good Research Practices Task Force [1, 2]: II-2, II-4

A discrete event simulation (DES) model was constructed, during COVID-19, to analyse how infection control measures, and possible infection related staff absence impact patient flow and efficient delivery of radiotherapy care.

Specific parts of the paper which defined the health-related decision problem, include:

- Abstract
- Introduction→Context
- Introduction→Radiotherapy simulation literature
- Materials and Methods→Study aim
- Materials and Methods→Study design

Specific parts of the Radiotherapy model STRESS documentation which defined the health-related decision problem, include:

- 1.1 Purpose of the model
- 1.3 Experimentation Aims

2. Is the modelled healthcare setting/health condition clarified?

… the physical context/scope (e.g., a certain healthcare unit or a broader system) or disease spectrum simulated was described.

ISPOR-SMDM Modelling Good Research Practices Task Force [1, 2]: II-2

A generic DES model was developed to investigate potential patient flow impacts of infection control measures and infected related staff absences. In this case the model was applied to a private German radiotherapy centre, where radiotherapy was used to treat cancer patients.

Specific parts of the paper which defined the healthcare setting and health care condition, include:

- Abstract
- Introduction→Context
- Introduction→Radiotherapy simulation literature
- Materials and Methods→Study aim
- Materials and Methods→Study design

Specific parts of the Radiotherapy model STRESS documentation which defined the healthcare setting and health care condition, include:

- 1.1 Purpose of the model
- 1.3 Experimentation Aims

3. Is the model structure described?

… the model's conceptual structure was described in the form of either graphical or text presentation.

ISPOR-SMDM Modelling Good Research Practices Task Force [1, 2]: II-3, II-6; VII-1

The model's conceptual structure, the flow of patients through a German Radiotherapy Centre, was defined in graphical and textual form.

Specific parts of the paper which defined the model structure, include:

- Materials and Methods→Model specification
- Figure 1, Figure 2, Table 3, Figure 3, Table 4, Table 5, Table 6

Specific parts of the Radiotherapy model STRESS documentation which defined the model structure, include:

- 2.1 Base model overview diagram
- 2.2 Base model logic
- 2.5 Components

4. Is the time horizon given?

… the time period covered by the simulation was reported.

ISPOR-SMDM Modelling Good Research Practices Task Force [1, 2]: II-2, II-3b

The model time horizon was 10 hours (08:00 to 18:00) plus a 30-minute grace period (18:00-18:30). These were the opening times of the Radiotherapy Centre and plus a grace period to process patients in the Centre.

Specific parts of the paper which defined the time horizon, include:

- Experimental design→Time horizon

Specific parts of the Radiotherapy model STRESS documentation which defined the run length (time horizon), include:

- 4.2 Run length

5. Are all simulated strategies/scenarios specified?

… the comparators under test were described in terms of their components, corresponding variations, etc

ISPOR-SMDM Modelling Good Research Practices Task Force [1, 2]: II-2e, II-3a

The 21 simulated scenarios used in our scenario-based analysis are named, and their input parameters are defined.

Specific parts from the paper which defined the simulated strategies/scenarios, include:

- Experimental design→Scenarios
- Table 4 and Table 6.

Specific references from the Radiotherapy model STRESS documentation which defined the simulated strategies/scenarios, include:

- 2.2 Base model logic
- 2.3 Scenario logic

6. Is the target population described?

… the entities simulated, and their main attributes were characterized.

ISPOR-SMDM Modelling Good Research Practices Task Force [1, 2]: II-2c

The target population, patients requiring radiotherapy and their key patient attributes (type, age) are defined. Additional parameters, and more detail are provided in the Radiotherapy model STRESS documentation.

Specific parts of the paper which defined the target population, include:

- Introduction→Context
- Introduction→Process of radiotherapy
- Introduction→Radiotherapy and COVID-19
- Materials and Methods→Study aim
- Materials and Methods→Study design
- Materials and Methods→Model specification
- Experimental design→Input parameter
- Table 4

Specific parts of the Radiotherapy model STRESS documentation which defined the target population, include:

- 2.5.1 Entities
- Table 4
- 3.3 Input parameters

Parameterisation and uncertainty assessment

7. Are data sources informing parameter estimations provided?

… the sources of all data used to inform model inputs were reported.

ISPOR-SMDM Modelling Good Research Practices Task Force [1, 2]: VII-1; VI-5

The data sources for key patient attributes (type, age) and human resource attributes are stated in the paper and in the Radiotherapy model STRESS documentation. The STRESS documentation contains information about arrival and service times. Estimates provided by stakeholders were used to create service (process) time distributions.

Specific parts of the paper which provided information about the data sources informing parameter estimations, include:

- Experimental design→Input parameter
- Table 4

Specific parts of the Radiotherapy model STRESS documentation which provided information about the data sources informing parameter estimations, include:

- 3.1 Data sources
- Table 10, Table 11, Table 12, Table 13, Table 14.

8. Are the parameters used to populate model frameworks specified?

… all relevant parameters fed into model frameworks were disclosed.

ISPOR-SMDM Modelling Good Research Practices Task Force [1, 2]: VI-4, VI-10

The paper includes key patient attributes (type, age) and human resource attributes. A complete list of input parameters including arrival patterns, and process service times are provided in the Radiotherapy model STRESS documentation.

Specific parts of the paper which specified the parameters to populate the model framework, include:

- Experimental design→Input parameter
- Table 4

Specific parts of the Radiotherapy model STRESS documentation paper which specified the parameters to populate the model framework, include:

- 2.1 Base model overview diagram
- 2.2 Base model logic
- 2.5 Components

9. Are model uncertainties discussed?

… the uncertainty surrounding parameter estimations and adopted statistical methods (e.g., 95% confidence intervals or possibility distributions) were reported.

ISPOR-SMDM Modelling Good Research Practices Task Force [1, 2]: VI-1, VI-7, VI-8, VI-9, VI-14

Uncertainty around key patient attributes (type, age), arrival and service times, and human resource attributes is discussed.

Additionally, Results are provided with 95% confidence intervals in all Box plots.

Specific parts of the paper which discussed model uncertainties, include:

- Materials and Methods→Verification and validation
- Experiment design→Multiple runs

Specific parts of the Radiotherapy model STRESS documentation which discussed model uncertainties, include:

- 4.3 Estimation approach
- Table 12, Table 13, Table 14, Figure 5, Figure 6

10. Are sensitivity analyses performed and reported?

… the robustness of model outputs to input uncertainties was examined, for example via deterministic (based on parameters' plausible ranges) or probabilistic (based on a priori-defined probability distributions) sensitivity analyses, or both.

ISPOR-SMDM Modelling Good Research Practices Task Force [1, 2]: VI-12

Extensive sensitivity analysis is undertaken and is provided in appendix S1→Model and validation folder online, which contains the model and data validation/sensitivity spreadsheets.

Specific parts of the paper which discussed and reported sensitivity analyses, include:

- Materials and Methods→Scenarios
- Materials and Methods→Verification and validation
- Results

Specific parts of the Radiotherapy model STRESS documentation which discussed and reported sensitivity analyses, include:

- 3.3 Input parameters
- Table 11, Table 12, Table 13, Table 14, Figure 5, Figure 6.
- 4.3 Estimation approach

Specific parts of S1→Model and validation which discussed and reported sensitivity analyses, include:

- Model and validation→validation.xlsx (several tests)
- Tests 1 and 2 were conducted but not included Mendeley. The multiple measures used to record waiting times, process/service times and length of stay reconciled. The input data to the model remained unaltered throughout the simulation runs.

- Tests 3 to 24 were repeated with COVID, with low-COVID and with high-COVID, which results in the 60 tests reported. JV to investigate the results to ensure they're correct.
- The numbers in brackets denote the reference in the validation.xlsx sheet on Mendeley.
- Test 1. Check time measurement (1) for 10 runs with/without COVID, as time measures captured using a) time measure start/end blocks, and b) via patient variables. Not reported see second point.
- Test 2. Check that the model inputs match model inputs extracted from the simulation model (2) for 10 runs with/without COVID. Not reported see second point.
- Test 3. Check the oncology appointments distribution matches the simulated oncology appointments distribution (3.1)
- Test 4. Check the number of CT and fraction patients specified matches the simulated CT and fraction patients (3.2)
- Test 5. Check the treatment type pathway distribution matches the model generated treatment type pathway distribution (3.3)
- Test 6. Extreme value test (4.1.1). Check model results if oncologists reduced to 0, base case = 2.
- Test 7. Extreme value test (4.1.2). Check model results if RTT reduced to 0, base case = 6.
- Test 8. Extreme value test (4.1.3). Check model results if MPE reduced to 0, base case = 1.
- Test 9. Extreme value test (4.1.4). Check model results if Administrators reduced to 0, base case = 2.
- Test 10. Extreme value test (4.1.5). Check model results if LINACS reduced to 0, base case = 2.
- Test 11. Extreme value test (4.1.6). Check model results if CTs reduced to 0, base case = 1.
- Test 12. Extreme value test (4.2.1). Check model results if oncologists increased to 5, base case = 2.
- Test 13. Extreme value test (4.2.2). Check model results if RTT increased to 10, base case = 6.
- Test 14. Extreme value test (4.2.3). Check model results if MPE increased to 5, base case = 1.
- Test 15. Extreme value test (4.2.4). Check model results if Administrators increased to 5, base case = 2.
- Test 16. Reduced oncologist appointment slot intervals and variability (4.3.1), to 20 minutes. Base case triangular (25, 30, 35) minutes. Center more congested earlier.
- Test 17. Reduced CT appointment slot intervals and variability (4.3.2), to 15 minutes. Base case triangular (25, 30, 35) minutes. Center more congested earlier.
- Test 18. Reduced Fraction (1st and subsequent) appointment slot intervals and variability (4.3.3), to 7 minutes. Base case triangular (8,10,12) minutes. Center more congested earlier.
- Test 19. Trace 1 oncology patient (4.4.1.1) through the system, main.enter_arrivals_onc = 1; main.enter_arrivals_CT = 0; main.enter_arrivals_fraction=0.
- Test 20. Trace 1 CT patient (4.4.1.2) through the system, main.enter_arrivals_onc = 0; main.enter_arrivals_CT = 1; main.enter_arrivals_fraction=0.
- Test 21. Trace 1 fraction patient (4.4.1.3) through the system, main.enter_arrivals_onc = 0; main.enter_arrivals_CT = 0; main.enter_arrivals_fraction=1.
- Test 22. Increase service time (4.5.1.1) fraction (not first) duration increases by 20%, main.duration_Fraction = 1.2 * triangular (3, 7, 10) minutes
- Test 23. Increase service time (4.5.1.2) fraction (not first) duration increases by 50%, main.duration_Fraction = 1.5 * triangular (3, 7, 10) minutes
- Test 24. Increase the variability of the first fraction (4.5.2) uniform (15,25). Base case = uniform (14,16)

Validation

11. Is face validity evaluated and reported?

… it was reported that the model was subjected to the examination on how well model designs correspond to the reality and intuitions. It was assumed that this type of validation should be conducted by external evaluators with no stake in the study.

ISPOR-SMDM Modelling Good Research Practices Task Force [1, 2]: VII-3

The lead author developed the model and conducted the initial analysis. The lead author conducted face validity tests with system stakeholders. The second author was brought into the project later and validated the model independently, before discussing the model with the lead author, and the other authors.

Specific parts of the paper which evaluated and reported face validity, include:

- Materials and Methods→Verification and validation

Specific parts of the Radiotherapy model STRESS documentation which evaluated and reported face validity, include:

- 1.2 Model Outputs
- Table 2
- 2.5.1 Entities
- Table 4
- 2.5.4 Queues
- Table 7
- 2.5.5 Entry/Exit Points

Specific parts of S1→Model and validation which evaluated and reported face validity, include:

- See response to 10.

12. Is cross validation performed and reported

… comparison across similar modelling studies which deal with the same decision problem was undertaken.

ISPOR-SMDM Modelling Good Research Practices Task Force [1, 2]: VII-5

Cross validation was not performed as a generic model such as ours has not been created. Existing models have been applied in other non-German contexts which differ from ours.

Specific parts of the paper which reported on cross validation, include:

- Materials and Methods→Verification and validation

13. Is external validation performed and reported?

… the modeler(s) examined how well the model's results match the empirical data of an actual event modelled.

ISPOR-SMDM Modelling Good Research Practices Task Force [1, 2]: VII-6, VII-7

External validation was performed and reported. As the model was created to respond to a situation in which infection control measures were necessary only the base case was externally validated.

Specific parts of the paper which reported on external validation, include:

- Materials and Methods→Verification and validation

Specific parts of the Radiotherapy model STRESS documentation which reported on external validation, include:

- 1.2 Model Outputs
- Table 2
- 2.5.1 Entities
- Table 4
- 2.5.4 Queues
- Table 7
- 2.5.5 Entry/Exit Points

Specific parts of S1→Model and validation which reported on external validation, include:

- See response to 10.

14. Is predictive validation performed or attempted?

… the modeler(s) examined the consistency of a model's predictions of a future event and the actual outcomes in the future. If this was not undertaken, it was assessed whether the reasons were discussed.

ISPOR-SMDM Modelling Good Research Practices Task Force [1, 2]: VII-11

Predictive validation was not performed as it was outside the scope of the collaboration. The timeline of the modelling project was short, 3 months, and as such external validity and other validity test were performed. This is a key area for future research.

Specific parts of the paper which reported on predictive validation, include:

- Materials and Methods→Verification and validation

Generalisability and stakeholder involvement

15. Is the model generalisability issue discussed?

… the modeler(s) discussed the potential of the resulting model for being applicable to other settings/populations (single/multiple application).

ISPOR-SMDM Modelling Good Research Practices Task Force [1, 2]: II-5

The model was designed using generic principles. This was to make it generalisable in the first instance to standalone German Radiotherapy Centres, but also to Radiotherapy Centres in other countries/contexts.

Specific parts of the paper which discussed generalisability, include:

- Materials and Methods→Model specification

- Discussion→Interpretation and further work

16. Are decision makers or other stakeholders involved in modelling?

… the modeler(s) reported in which part throughout the modelling process decision makers and other stakeholders (e.g., subject experts) were engaged.

ISPOR-SMDM Modelling Good Research Practices Task Force [1, 2]: II-1

Stakeholders were involved with conceptualising the model, providing data, verifying and validating the model and suggesting policies (scenarios) to investigate.

Specific parts of the paper which discussed the involvement of decision makers or other stakeholders, include:

- Materials and Methods→Data sources
- Materials and Methods→Verification and validation
- Experimental Design→Input parameter (Table 4)

Specific parts of the Radiotherapy model STRESS documentation which discussed the involvement of decision makers or other stakeholders, include:

- 1.3 Experimentation Aims
- 3.1 Data sources
- Table 10
- Figure 5

17. Is the source of funding stated?

… the sponsorship of the study was indicated.

ISPOR-SMDM Modelling Good Research Practices Task Force [1, 2]: VII-1

The authors reported any funding relevant to the research in the acknowledgment section and via the journal submission system.

Specific parts of the paper which stated the funding source, include:

- Acknowledgements

18. Are model limitations discussed?

… limitations of the assessed model, especially limitations of interest to decision makers, were discussed.

ISPOR-SMDM Modelling Good Research Practices Task Force [1, 2]: VII-1

All models have strengths and weaknesses being simplifications of reality.

Specific parts of the paper which discussed the strengths and limitations, include:

- Materials and Methods→Data sources
- Materials and Methods→Assumptions

- Discussion→Strengths and limitations

Specific parts of the Radiotherapy model STRESS documentation which discussed the strengths and limitations, include:

- 3 Data

References

1. Caro, J. J., Briggs, A. H., Siebert, U., Kuntz, K. M., & ISPOR-SMDM Modeling Good Research Practices Task Force. (2012). Modeling good research practices--overview: a report of the ISPOR-SMDM Modeling Good Research Practices Task Force–1. *Medical Decision Making*, *32*(5), 667-677. https://doi.org/10.1177/0272989X12454577

2. Caro, J. J., Briggs, A. H., Siebert, U., Kuntz, K. M., & ISPOR-SMDM Modeling Good Research Practices Task Force. (2012). Modeling good research practices--overview: a report of the ISPOR-SMDM Modeling Good Research Practices Task Force–1. *Value Health*, *15*(6), 796-803. https://doi.org/10.1016/j.jval.2012.06.012

3. Mielczarek, B., & Uzialko-Mydlikowska, J. (2012). Application of computer simulation modeling in the health care sector: a survey [Article]. *Simulation-Transactions of the Society for Modeling and Simulation International*, *88*(2), 197-216. https://doi.org/10.1177/0037549710387802

4. Günal, M. M., & Pidd, M. (2010). Discrete event simulation for performance modelling in health care: a review of the literature. *Journal of Simulation*, *4*(1), 42-51. https://doi.org/https://doi.org/10.1057/jos.2009.25

5. Katsaliaki, K., & Mustafee, N. (2011). Applications of simulation within the healthcare context. *Journal of the Operational Research Society*, *62*(8), 1431-1451. https://doi.org/10.1057/jors.2010.20

6. Fone, D., Hollinghurst, S., Temple, M., Round, A., Lester, N., Weightman, A., Roberts, K., Coyle, E., Bevan, G., & Palmer, S. (2003). Systematic review of the use and value of computer simulation modelling in population health and health care delivery. *Journal of Public Health Medicine*, *25*(4), 325-335. https://doi.org/https://doi.org/10.1093/pubmed/fdg075

7. Jun, J. B., Jacobson, S. H., & Swisher, J. R. (1999). Application of discrete-event simulation in health care clinics: A survey. *Journal of the Operational Research Society*, *50*(2), 109-123. https://doi.org/https://doi.org/10.1057/palgrave.jors.2600669
