# Supplementary material for: Evaluating the impact of COVID-19 protection measures and staff absence on radiotherapy practice: A simulation study
Source: PLoS One. 2025 Jan 16;20(1):e0314190. doi: 10.1371/journal.pone.0314190 (PMC11737702; doi:10.1371/journal.pone.0314190)
Supplement: S3 Appendix — A check list of verification and validation tests performed. This document supplements the 1. Model and Validation and 2. STRESS documentation from S1 and S2 Appendices. (PDF) [file pone.0314190.s003.pdf]

Simulation model verification and validation

Verification – Building the model correctly: compare conceptual and simulation models

Validation – Building correct model: compare model and real system

Validation is not an either/or proposition, _*no model is ever truly valid*_. Each model iteration involves some cost, time, and effort. The modeller must weigh the possible, but not guaranteed, increase in model accuracy versus the cost of increased validation effort (Banks 2014, p394).

The _*purpose, or objectives, of a model must be known before it can be validated*_ (Robinson, 2014). This purpose may have been determined at the start of the simulation study, being expressed through the objectives.

Law (2014) defines an assumption document, as useful to document all model concepts, assumptions, algorithms, and data summaries to improve validation, and enhance the credibility of the model. An assumptions document is also known as a conceptual model (Law 2014). This is similar to the STRESS guidelines (Monks et al 2018) and project specification (Robinson 2014).

The second column of the Table on page 2 outlines key verification and validation techniques from six referenced textbooks. The third column indicates whether these techniques were used and provides references to sections of the paper, presentation, or existing model documentation where their application is described.

References

1. Banks, J. (2014). **Discrete-event system simulation** (5th ed., p. 640). Pearson.
2. Hillier, F. S., & Lieberman, G. J. (2021). **Introduction to operations research** (Eleventh edition, International student edition.). McGraw-Hill Education.
3. Law, A. M. (2015). **Simulation modeling and analysis** (5th ed., p. XVIII, 776). McGraw-Hill Education.
4. Pidd, M. (2004). **Computer simulation in management science** (5th ed., p. XVI, 311). Wiley.
5. Robinson, S. (2014). **Simulation: the practice of model development and use** (2nd ed.). Palgrave Macmillan.
6. Sterman, J. D. (2000). **Business dynamics : systems thinking and modeling for a complex world** (p. XXVI, 982). Irwin McGraw-Hill.
7. Monks, T., Currie, C. S. M., Onggo, B. S., Robinson, S., Kunc, M., & Taylor, S. J. E. (2018). Strengthening the reporting of empirical simulation studies: Introducing the STRESS guidelines. *Journal of Simulation*, *13*(1), 55-67. https://doi.org/10.1080/17477778.2018.1442155

V&V	Validation/Verification (References)	Notes and references to existing model documentation and paper sections
Verification building the model correctly	1. **Compare conceptual** and **simulation** models [1, 3, 4, 5, 6]	Yes. Stakeholders confirmed that Figure 1 and Figure 2 were modelled correctly.
	2. Are **input parameters** represented **correctly**? [1, 3, 4, 5, 6]	Yes. Authors 1 and 2. Checked that the input data were used in the model.
	3. Is **logical model structure** represented **correctly**? [1, 3, 4, 5, 6]	Yes. Author 1 checked the model structure with system stakeholders.
	4. Engage people familiar with the system. **Experts. Structured walk through** [1, 2, 3, 4, 5, 6]	Yes. Stakeholders confirmed that Fig 1 and Fig 2 were modelled correctly.
	5. Get **another simulation expert to check** the simulation model. [1, 4, 5, 6]	Yes. Author 2, more experience modeller, checked the author 1's model.
	6. Create **flow diagram** which includes logical system actions & follows model logic. [1, 3, 4, 5, 6]	Yes. See Fig 1 and Fig 2. The AnyLogic model is made available online.
	7. Examine **model output for reasonableness** under a variety of input settings. [1, 2, 3, 4, 5, 6]	Yes. Initial model experimentation, scenario analysis, and validation tests.
	8. If **animation** is used verify the model logic with the animation. If graphs are considered* [1, 2, 3, 4, 5, 6]	Yes. (Individual) patients tracked through the system, using floor plans and graphs
	9. **Debugging** (interactive run controller) step through model, **trace** individual entities. [1, 2, 3, 5, 6]	Yes. Patients tracked through system with set parameters to validate routing
	10. **Graphical interface** and **documentation** for verification and validation [1, 2, 3, 4, 5, 6]	Yes. Graphical, tabular and animation, along with the software's GI were utilised.
Validation building correct model<br>*Calibration: iterative model vs. real system comparison*	11. Attempts to **confirm a model** is an **accurate representation** of the **real system**. [1, 2, 3, 4, 5, 6]	Yes. Through discussion with stakeholders
	12. **Model calibration**. Use **difference between model** and the real **system**, to improve the model. [1, 3, 5, 6]	Yes. For each mode iteration (version) input parameters were evaluated.
	13. Subjective tests: system **stakeholders, judge** the model & its output(s) & judge if model is accurate enough. Use **SSM** and **DOE**. **Turing Test(s)**, Structural assumptions. **Face validity** [1, 2, 3, 4, 5, 6]	Yes. Subjective comparison of service times, patient distributions, arrival patterns etc.
	14, Objective tests. **Statistical test(s) compare an aspect(s) of the system with the model** output data. If **purpose** changes, revalidate in terms of relevant response(s). [1, 2, 3, 4, 5, 6]	Not tested.
	15. **Iterative** process. **Comparing model/system** and revising the **conceptual/operational models** to accommodate perceived model deficiencies until model deemed accurate. [1, 3, 4, 5, 6]	Yes. The model was developed in an iterative manner. Eight versions of the model were created.
	16. **Involve model users** in model construction (conceptualization→implementation), to build an adequate degree of realism into the model (reasonable **assumptions** & **data**). [1, 3, 4, 5, 6]	Yes. Stakeholders were involved in conceptualisation, provided estimations for data distributions, and in the verification and validation of the model.
	17. **Sensitivity analysis** (check face validity). Model user asked if it **behaves in the expected way when an input variable(s) is changed**. **Extreme value tests?** [1, 2, 3, 4, 5, 6]	Yes. Extensive sensitivity analysis was undertaken see Model and validation→validation.xlsx
	18. **Data** assumptions: based on the collection of reliable data. Data reliability: **statistical tests for homogeneity of data**. Goodness-of-fit test(s): chi-square, Kolmogorov–Smirnov test, t-Tests, distribution-free tests, *bootstrapping*, graphical methods, **confidence intervals,** % difference, correlation coefficient, *regression analysis*, Kruskal-Wallis test of homogeneity of populations, Mann-Whitney, *time series methods (spectral-analysis)*, *correlated inspection approach*, Welch, P-P plots [1, 3, 4, 5, 6]	No. System stakeholders and the literature were used to determine distributions and model input values; therefore, the model data were not statistically evaluated.
	19. Can the model **predict future behaviour** of the system (inputs: data=real). Model should be accurate enough to make good predictions, for a range of input data sets. [1, 3, 4, 5, 6]	Not tested. Could be the subject of future research.
	21. **Input-Output Validation**: Use **Historical Input Data**, alternative to generating input data, to drive the model and compare model output with system data. *Black box* [1, 3, 4, 5, 6]	Yes. In terms of the number of arrivals. Service times. Patient characteristics.
	22. Validate against **another** (simulation/analytical) **model** [2, 3, 5, 6]	Not tested. No similar German private Radiotherapy Centre model available.
	23. Subjective tests: **Modeller experience** and intuition about complex systems. [3, 5, 6]	Yes. Author 2, more experience modeller, checked the author 1's model.
